# Contextual Factors Among Indiscriminate or Large Attacks on Food or Water Supplies, 1946-2015

**DOI:** 10.1089/hs.2015.0056

**Published:** 2016-02-01

**Authors:** Julii Brainard, Paul R. Hunter

## Abstract

This research updates previous inventories of malicious attacks on food and water and includes data from 1946 through mid-2015. A systematic search of news reports, databases, and previous inventories of poisoning events was undertaken. Incidents that threatened or were intended to achieve direct harm to humans and that were either relatively large (more than 4 victims) or indiscriminate in intent or realization were included. Agents could be chemical, biological, or radionuclear. Reports of candidate incidents were subjected to systematic inclusion and exclusion criteria as well as validity analysis (not always clearly undertaken in previous inventories of such attacks). We summarize contextual aspects of the attacks that may be important for scenario prioritization, modelling, and defensive preparedness. Opportunity, and particularly access to dangerous agents, is key to most realized attacks. The most common motives and relative success rate in causing harm were very different between food and water attacks. The likelihood that people were made ill or died also varied by food or water mode and according to motive and opportunity for delivery of the hazardous agent. Deaths and illness associated with attacks during food manufacture and prior to sale have been fewer than those in some other contexts. Valuable opportunities for food defense improvements are identified in other contexts, especially food prepared in private or community settings.

## Abstract 2

This research updates previous inventories of malicious attacks on food and water and includes data from 1946 through mid-2015. The authors made a systematic search of news reports, databases, and previous inventories of poisoning events, including incidents that threatened or were intended to achieve direct harm to humans and that were either relatively large (more than 4 victims) or indiscriminate in intent or realization. Agents could be chemical, biological, or radionuclear. Opportunity, and particularly access to dangerous agents, is key to most realized attacks. The most common motives and relative success rate in causing harm were very different between food and water attacks. The likelihood that people were made ill or died also varied by food or water mode and according to motive and opportunity for delivery of the hazardous agent. Deaths and illness associated with attacks during food manufacture and prior to sale have been fewer than those in some other contexts.

Interest in food and water security has led to comprehensive inventories being generated in the past 20 years, to identify common factors in previous attacks that employed biological, chemical, or radionuclear agents. The most recent previous comprehensive inventory was completed in 2008.^1^ Previous inventories identified agents and types of food or water supplies attacked (useful for scenario modeling), and commonly reported summary statistics about location, total number of cases, type of food or water supply, and type of agent.^1-3^ Pressing vulnerabilities and risks facing water supply have been discussed,^4,5^ while Purver tried to summarize likely motives, organizations, and agents in future terrorist attacks.^3^ Mohtadi and Murshid used extreme value theory to predict that future events would be more frequent and affect more people.^6^ Multifaceted aspects of preparedness against terrorist attacks, or lack thereof, have been discussed as part of the inventories of previous events, usually in the context of the powers given to regulatory bodies and overall optimal national government strategy.^3,7^

This article updates the previous inventories of food and water attacks to include data through mid-2015. Reports of candidate incidents were subjected to systematic inclusion and exclusion criteria as well as validity analysis (not always clearly undertaken in previous reports). We report on contextual aspects of the attacks that may be important for scenario prioritization, modeling, and disaster preparedness but that have not been identified previously. In particular, the types of opportunities or motives most often associated with threat or deaths are identified, and possible protection measures are discussed.

## Methods

### Search Strategy

Wherever possible, scientific articles, court reports, and primary source news reports were sought and preferred for citation of individual incidents rather than reviews or inventory documents, but some original reports were unavailable.

General searches for scientific articles (using PubMed and Scopus) were undertaken. We used the search terms *intentional poisoning* or *deliberate poisoning* in the title field, and the following search string (OVID gateway): ((food* or water*).tw. or exp water/ or exp ice/ or exp beverages/ or exp food/ or exp drink/) and (poison* or contaminat* or attack)).tw. Almost no eligible reports were found using these search phrases. Searching without the title words (.tw.) restriction led to a large number (many thousands) of mostly irrelevant hits, which we lacked the resources to screen for eligibility. However, using details extracted on agent and location from news accounts and other sources found via other search strategies (see below and data extraction section), we again searched PubMed and Scopus for scientific reports on individual incidents and found some case reports not cited elsewhere.

Because relatively few attacks on food or water supplies are described in scientific (peer-reviewed) literature, to inventory such incidents from unclassified data in the inclusive dates 1946 to July 23, 2015, we employed search methods and accessed sources as follows.

**Figure 1.**
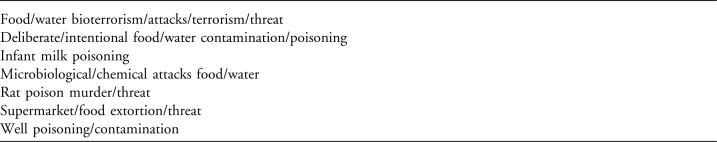
Phrases and Terms Used in Internet Searches. Alternatives are indicated by slashes. For instance, “Rat poison murder/threat” means both phrases “rat poison murder” and “rat poison threat” were put into the internet search engine.

An internet search engine was used to find books (Google Books), grey literature (all Google databases), news media reports (Google News), and scholarly articles (Google Scholar) that matched English language search terms below (Figure 1). The phrases in Figure 1 were chosen considering the articles found after scoping searches using the simpler phrases *food attack* and *water attack.* The first 4 pages of search results (80 hits) were screened for eligible incidents or documents that inventoried deliberate poisoning incidents. News media reports were searched both with no date limits and again specifically for publication in individual single years in the period 2004 to 2015 (the period most weakly covered by previous inventories of food and water attacks). Reports that were inventories of previous attacks on food and/or water, and meant to be comprehensive within their stated eligibility criteria, were hand-checked for references to further inventories, relevant databases, and individual incident reports. These inventories and any specific databases that they cited (see supplemental file S1 at www.liebertonline.com/hs) were searched for eligible attacks within our date limits.

### Eligibility

This inventory is for events that were indiscriminate in intention or realization or that affected a relatively large number (more than 4) of specific targeted individuals. The rationale for omitting smaller events is to exclude many events that were very limited in scope, impact, or public health response. Closely linked small attacks, such as a series of single poisonings by 1 individual or that resulted from the coordinated actions of 2 or more people, were grouped and treated as a single incident with regard to eligibility criteria and summary statistics.

Adult suicides were not eligible, but poisoning of groups (more than 4) of targeted children (under 16 years old) as part of group suicide events were treated as murder and therefore eligible.

Events from January 1, 1946, through July 23, 2015, were included. There were no geographical restrictions. Fluent speakers were consulted or translation software was used where reports were not available in English.

The event had to feature deliberate adulteration of food, beverage, or water items, with intention (real or threatened) to cause harm to other humans. Throughout this article we use the term *food* as an umbrella term that also includes beverages and any other edible substance that is not a source for plain potable water. Deliberate attack (rather than causing harm by negligence) must have been specified by police, government bodies, identified witnesses, or a credible party. Exposure to a poisonous agent via inhalation, injection, or other non–oral ingestion means was not eligible, even when linked to an event that involved poisoning via food or water.

An unrealized but threatened attack must have evidence of intent to be perceived as a genuine threat. Thus, we excluded candidate threats that were mere rumors, an isolated single threat with no specifics of means or target, or an individual stating general interest in the mechanics of poisoning people. Examples of threats accepted as expressing genuine intent (at least at the point when the threat was issued) were: widely published claims of responsibility, threats made with specifics of reason why, and method or evidence of a criminal investigation to verify that a plot existed.

Agents included any substance that could in theory be dissolved (invisible to the naked eye) into the target food, drink, or water source. Thus, biological, chemical, and radionuclear agents were included, as could be very finely powdered physical agents such as ground glass or metals, as long as the reason for the adulteration was intention to cause harm to humans.

### Data Extraction

Extracted data were bibliographic details of source document(s), location(s) and date of attack(s), type of food or water supply attacked, contaminant, person(s) responsible with motives, intended target(s), numbers ill or killed, and public health or criminal investigation response and outcomes.

### Validity Analysis

**Figure 2.**
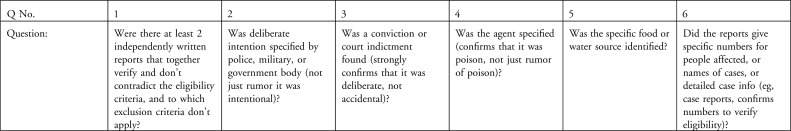
Validity Questions for Food and Water Attack Inventory. Scores for each answer: No = −1, Yes = 1, except score = 2 for “Yes” to Question 3, conviction or indictment. Score = 0 when answer = partly/not applicable/unclear.

Validity questions are shown in Figure 2 and were intended to evaluate the strength of evidence that confirms eligibility criteria for each alleged incident rather than validating individual sources about each event. “Yes” answers were preferred for each question. A score was assigned to each incident based on the validity assessment for reports about that event, with scores of No = –1, unclear or partly/not applicable = 0, and Yes = 1. The question of whether a conviction or indictment was documented scored 2 for a Yes answer, because this aspect of validity, whether harm was intended, was often the most uncertain of our validity questions.

Scores for all validity questions were summed to give a combined score for the strength of evidence for each event. The maximum theoretical score was 8; the minimum possible theoretical score was −7. Where an event scored 5+, it was deemed to be a highly credible food or water attack. Scores of 3 or 4 were interpreted as a credible event. Scores from 0 to 2 (inclusive) were considered to be an event with weak evidence; usually, some but not all aspects of eligibility were well confirmed. Items with scores below 0 were interpreted to have poor evidence of credibility, which again is more often due to lack of evidence than poor quality of evidence.

## Results

Nine previous large inventories of poisoning attacks on water or food were found.^1-9^ Together they indicated a large number of source databases to search (listed in supplemental file S1). All incident reports were checked for our inclusion and exclusion criteria, using original news or scientific reports wherever possible rather than information in a review. Our full inventory of eligible food and water attacks is available as supplemental data (see S2, www.liebertonline.com/hs).

### Water

We found 84 incidents of actual or threatened water supply poisoning: 21 (25%) reports were in Europe, 22 (26%) in the United States, 4 (4.8%) were elsewhere in the Americas, 10 (12%) in the Middle East, and 8 (9.5%) in Africa, with the remainder occurring in other parts of the world. Specific agents used in water attacks are listed in Table 1. The nature of the agent tends to be vaguer in water attacks than in food events and was not clearly specified in 23 incidents (27%). Chemical agents dominate both in number of implicated attacks and associated deaths or illness.

**Table 1.** Specific Agents Used in Water or Food Attacks, 1946-2015

*Agent*	*No. of Total Attacks*	*Most Common Motive(s), (no. of attacks)*	*Linked Deaths, Total*^a^	*Ill Survivors, Total*^a^
**Agents in Water**				
**Chemical**				
Arsenic	1	Unclear	0	5
Cyanide	5	Political (5)	5	2
Herbicide, pesticide, or insecticide	11	Unclear (5) Political (4)	20	361
Other specific single or multiple agents	17	Unclear (8) Political (4)	2	149
Mercury	5	Political (1) Unclear (1)	0	0
Nerve gases (eg, VX)	3	Political (2)	0	5
Ricin	2	Multiple (1) Labor dispute (1)	0	0
**Microbiological**				
Cholera	2	Political (2)	0	Unclear
Dead bodies or feces	4	Political (3)	0	9
Other microbiological or organic contaminants	11	Political (6)	0	2
**Radionuclear** (plutonium)	1	Not financial extortion	0	0
**Agents in Food**				
**Chemical**				
Arsenic	11	Personal malice (3) Unclear (3)	309	842
Cleaning fluids	5	Extortion (2)	0	76
Cyanide-based	20	Extortion (6) Unclear (6)	286	6
Herbicide, pesticide, or insecticide	26	Unclear (8) Extortion (6)	10	3,138 ^b^
Rodenticide (clearly tetramine)	15	Business rivals (6) Unclear (5)	59	985
Strychnine, including in rodenticides	3	Unclear (2)	0	2
Rodenticide (all others, including thallium)	37	Unclear (11) Personal malice (8)	103	1,096
Specific others or agents in multiple categories	41	Extortion (11) Unclear (11), Political (7)	35	365
Prescription drugs	7	Unclear (3) Personal Malice (2)	1	29
Ricin	2	Political (1) Extortion (1)	0	0
**Microbiological**				
HIV-AIDS	4	Extortion (4)	0	0
Other microbiological/ organic contaminant	13	Extortion (4) Political (4)	0	912
**Radionuclear** (iodine-125)	1	Love-life related (1)	0	0

Totals add to <84 (water) and <224 (food) due to vaguely specified agents in many incidents.

^a^ These estimates of how many people were made ill by each type of agent are probably conservative, because total numbers of dead or ill were often unclearly specified, although it is also likely that some deaths or illnesses were inaccurately linked to attacks. Reports were not specific about the number made ill in some attacks (*n* = 28 food, *n* = 11 water).

^b^ 2,843 people in Japan were reported ill due to contamination in imported food from China in a single event (blamed on a disgruntled factory employee).^11-15^

Fifty-five events (65%) were attacks against community drinking water; 8 (9.5%) were attacks on drinking supplies for police, military, or refugees; and 17 (20%) incidents involved targeting of other limited groups (such as neighbors, children in a nursery, or workers in a specific office). Five attacks were on bottled water. Primary motives were political (*n* = 45, 54% of incidents), unclear (*n* = 27, 32%), financial (usually extortion, *n* = 5, 6%), labor disputes (*n* = 3, 3.6%), or other (*n* = 5, 6%).

#### Threats Versus Realization

The impacts of the actual or threatened water poisoning (whether anyone ingested the contaminant) were unclear in 8 (9.5%) incidents. There was no evidence of an actual plan or attempt to contaminate water in 23 of the 84 (27%) incidents: These seemed to be threats only (most often for financial extortion). Of the 84 possible water attacks, 41 (49%) were clearly unsuccessful (no ingestion). Of 35 clearly realized attacks (where someone drank contaminated water), only 2 occurred after explicit warnings (both extortion attempts).

Illness from ingestion but without deaths was a feature of 18 incidents, while actual deaths from ingestion were reported in 7 incidents (8.3%). The number of deaths in fatal attacks was fewer than 6 persons in 5 of these incidents. However, 19 people died when poisoned water (and confectionary) were given to police recruits in the Philippines in 1987 for unknown reasons,^5,6^ and more than 200 people are believed to have died in a series of attacks on community water supplies, alleged to be politically motivated and perpetuated by the Rhodesian government against insurgent communities in 1978 to 1980.^10^

Compared to food attacks (see subsequent discussion), water attacks are much more likely to be linked to violent political objectives, and this is especially true for realized attacks: 54% of all attacks had political motives, while 26% (9/35) of the realized water attacks occurred in places experiencing armed conflict.

### Food

A total of 224 food attacks were found: 53 (24%) reports described poisoning events in the United States or Canada, 44 (19.6%) were in the People's Republic of China, 20 (8.9%) from elsewhere in the Far East, 8.9% in the Middle East, 23 (10.3%) in the UK, 25 (11.6%) elsewhere in Europe, and the remainder were in other parts of the world.

The most common motives were unclear (*n* = 57, 25% of events), financial extortion (*n* = 49, 22%), other financial (*n* = 22, 10%), political (*n* = 36, 16%), personal malice (*n* = 20, 9%), part of labor disputes (*n* = 11, 5%), and other (*n* = 27, 12%). A total of 1,171 deaths were confirmed from deliberate food poisoning in this inventory of (larger and indiscriminate) events. The motive of extortion led to just 1 death from food poisoning attacks in this inventory, while personal malice and business rival motives led to 48 and 49 deaths, respectively (combined they equal 8.2% of total deaths). Political motivations were responsible for 316 (27%) deaths, but the largest group by far were deaths associated with coercion and murder at mass (religious cult) suicide events, which accounted for at least 509 involuntary deaths (43.5% of total). Motives were unclear in 132 (11.3%) deaths, while other identified motives were linked to 116 (9.9%) deaths.

Agents used in food attacks are listed in Table 1. The vast majority of agents were purely chemical (*n* = 180, 80.3% of all food attacks, and *n* = 94, 73%, of 128 attacks in high-income countries). Microbiological or radionuclear agents featured in only 23 (10.3%) attacks. No deaths resulted from radionuclear agents, but biological agents were associated with about the same number of deaths as arsenic or tetramine. The agent was unclear in 29 (13%) attacks on food (many of these were unrealized threats, especially extortion). The totals in this breakdown are greater than 100% because combined chemical and biological agents were implied in some attacks. Among chemical agents, rodenticides were the most frequent (28% of all attacks), and cyanide-based products specifically account for 9% of attacks. Arsenic (5%), cleaning fluids (2%) and herbicides, insecticides, or pesticides were also common.

#### Threats Versus Realization

The majority of attacks in the food attack inventory (140, or 62%) were realized (someone ingested the harmful substance). In 94% of the realized 140, there was no threat warning issued beforehand. About a quarter of all 224 incidents (*n* = 60, 27%) were merely threats without realization; most of these were motivated by financial extortion. Of attempts or plans without accompanying threats, 14 (6.2%) were discovered before anyone ingested the dangerous contaminant. The extent to which a threat or plot was realized (whether anyone ingested the hazardous substance) was unclear in 9 events (4% of total).

A large variety of foods were targeted and at all points in the production chain, from field to factory to shop to plate(s). Most (*n* = 61, 73% of 84) threats or plots (without realization) were at points in manufacture or threats of adulteration of items on shop shelves. Eleven (13.3%) threats or plots (without realization) were targeted at restaurants or catered food. In contrast, most realized attacks were on food items supplied and prepared by volunteers, co-religionists, family, or catering staff (*n* = 78, 56%), and this is also the context for the vast majority of deaths (1,024, or 94% of total). After 1948, only 62 of 790 (7.8% of all) deaths from food attacks were in the context of paid preparation for ready-to-eat food (such as from a restaurant^1,6,11-21^), as opposed to volunteer or community/unpaid food preparation, and most (46 of 62) of these deaths associated with paying for prepared food were linked to business disputes in China.^12-19^

### Validity Assessment

The quality of eligible reports was variable. Validity attributes of reports did not differ substantially by type of target (food or water), so the validity assessment of food and water is discussed together. Eligibility of many incidents (*n* = 103, 46%) was confirmed by just 1 original report for most water or food incidents, often translated from a non-English language. The data from these incidents should be interpreted with caution. This is because for incidents with multiple source reports, it was common that some alleged deliberate intent while others suggested negligence.

Included events had deliberate intention to cause harm confirmed (or alleged) by police, military, or sovereign government sources (97%); in the other 30 incidents (3% of total), credible sources included medical professionals, representatives of large companies, truth commissions, and media statements by the would-be poisoners. A report of confession, conviction, or indictment to confirm deliberate intention to cause harm or threaten to cause harm was found for only 104 (33.7%) food or water attacks. However, it seems likely that many indictments and convictions are not widely reported, reflecting a bias in media coverage about newsworthiness.

The dangerous agent was less likely to be clearly specified in water attacks (29/84 = 35% of incidents) than food attacks (42/224 events, 19%). This partly reflects less-developed plots and threats that tend to be more associated with water than food attacks. In contrast, type of water supply tends to be very clearly stated in available reports (just 1 incident where water source was poorly identified), whereas the types of food attacked or threatened with contamination is more often unclear (35/224 incidents). Less than 9% of food or water attack events (27/308) had unspecific information with regard to how many people were made ill or killed (realized or threatened events). The numbers of people affected (especially those only made ill) sometimes varied between reports for understandable reasons: Our sources were media stories about developing events, mostly without medical confirmation of cause of illness.

Overall, there was better confirmation about food attacks than water attacks (see Table 2). Of reported food attacks, 31.2% were highly credible, compared to just 8.3% of water incidents. Just 24 (10.7%) food attacks were poorly confirmed, compared to 25 water events (30% of all identified water attacks). The poorer validity scores for water are partly because of the motives involved: Most water attacks were vaguely specified extortion attempts, and hence it was less likely that a specific agent was threatened or that the people responsible were identified and convicted.

**Table 2.** Validity Scores

*Interpretation*	*Validity Score*	*No. of Food Attacks*	*No. of Water Attacks*
Highly credible (7-5)	7	40	0
31.2% of food attacks	6	16	0
8.3% of water attacks	5	14	7
Credible (4-3)			
26.3% of food attacks	4	37	0
25.0% of water attacks	3	22	21
Weakly confirmed (2-0)	2	31	10
31.7% of food attacks	1	27	9
36.9% of water attacks	0	13	12
Poorly confirmed (−1 to −3)	−1	11	8
10.7% of food attacks	−2	9	12
29.8% of water attacks	−3	4	5
Summary	Average scores = 3.16 for food attacks 0.94 for water attacks	Σ = 224	Σ = 84

## Discussion

Many types of food were targeted and at every point in the production chain up to the moment of consumption. Water poisoning events were much more indiscriminate in intent and impact and much more likely to be politically motivated than attacks on food. Water attacks are also much less likely to be successful because of less specific planning and the large volumes of agent required to have a significant impact (due to heavy dilution).

Many threats were presented as genuine, but turned out to be only threats, and it seems they did not feature true desire to do harm to health; it is unusual that extortion leads to illness or mortality. This may be a small consolation to consumers but even less so to manufacturers and retailers, for whom commercial security still means treating every threat as genuine. Economic and environmental damage from attacks without ingestion can also be very high.^2,4,22-33^

When threatened harm was accompanied by an actual plan of action, attacks were least successful that targeted raw materials (the field), manufacturers, and retailers. This probably reflects the high security standards that the food production industry have embraced or have been required to implement in recent decades, as described by many standards documents or legislation (eg, BSI Standards Ltd^28^ and FSMA^34^).

The choice of dangerous agent sometimes reflected a “faddish” interest in sensationalized contaminants or traditional notoriety, such as arsenic and cyanide over many years, HIV-AIDS in the 1990s,^2,35,36^ or Ebola in 2014.^37^ Probable copycat attacks (threatened and realized) were also identified in our inventory.^38-45^ Mostly, however, the choice of agent seemed to be determined by availability (eg, rodenticides in household products,^13,46^ or infectious microbes among lab workers^2,47^), and most agents were chemical in nature. Li and colleagues^13^ argued that limiting public access to rodenticides had reduced the number of attacks using rat poison in China. Biological agents tend to be unusual, are more associated with threats than implementation, and are not as effective when deployed. Radionuclear agents were very uncommon in our inventory (just 2 of all 308 water and food attacks).

The events associated with highest mortality (and possibly highest illness rates as well) tended to be associated with political or religious motives (or both). Nonprofessional catering for specific targeted groups was a feature of the most successful attacks (ie, perpetuated by community volunteers, family members, or co-religionists).

As important as it is to protect against and prepare for malicious poisoning events, it is important to be aware that deaths and illness from *accidental* poisonings have affected many thousands more people than malicious deliberate events in the 1946-2015 period.^1,48^ Widespread accidental poisoning is also quite problematic because of the implications for identifying malicious poisoning events: Some apparent accidents may in fact have been deliberate events, masking true attacks.

Ideally, future research would include full analysis of the effectiveness of public health response to malicious food and poisoning events. Unfortunately, this is not yet practical because of the lack of published detailed and critical assessments of the public health responses in this type of event. Relatively few food or water attacks (*n* = 21) were at least partially documented in peer-reviewed scientific literature. There was no consistent systematic analysis of public health response in these publications, making it hard to draw meaningful conclusions. Most of the scientific or detailed reports concentrated on forensic investigation or successful treatment approaches rather than giving detailed accounts of public health actions to minimize further harm. Published and focused assessments of health protection failures in actual attacks are relatively rare (most are too small to generate sufficient public interest) and otherwise potentially sensitive (they could provide too much explicit information to would-be attackers about vulnerabilities).

One public health observation that we can make with confidence is that swift diagnosis of the causative agent is very valuable (especially in the case of realized attacks). Quick identification of the precise poison was credited for saving lives,^49^ while delayed recognition has led to unnecessary alarm^50^ or greater uncertainty about who was exposed and how.^51,52^ Early identification also supports accurate, timely, and effective communication about any possible wider threat to public health, which is very desirable following high-profile events.^53,54^

A strength of this report is in the rigor of inclusion and exclusion criteria, as well as the systematic validity analysis. Most previous inventories of food or water attacks did not describe a systematic methodology for verifying all eligibility criteria and did not (as clearly as we have) grade events by credibility of supporting evidence. The cause of poisoning in food or water can be very unclear, and it is not unusual in poisoning events for vehement accusations of criminal intent to be made which are later determined to be unfounded.^1,55,56^

### Limitations

Like similar previous efforts, it is not possible for this inventory to be perfectly complete. The quality of the evidence to verify eligibility criteria was not ideal; usually only 1 original report was available, which was produced for news media rather than scientific standards of evidence verification. It is also very likely that many threatened attacks on food and water are never reported in the public domain and therefore are not part of this inventory, but they may be listed in classified databases or known to specific private sector organizations.

A note of caution applies to our suggested links between likely fatality of attacks and context or motives, because the outcomes of some incidents (including fatalities) are often uncertain and may have been poorly recorded. This especially applies to historical events with political sensitivity, where adequate reporting may have been suppressed, such as, for example, attacks by the Brazilian government on indigenous populations from 1957 to 1965.^2,57^ In the Brazilian incidents, sugar was adulterated with arsenic and given to rural isolated communities among whom subsequent illness or fatalities were not recorded. Political motives were also implicated in claims^10^ that in 1978-1980 the Rhodesian government distributed thallium-contaminated meat to insurgents who then passed on the foodstuffs to hungry civilians, and government employees attempted to add cholera to water sources, with subsequent unknown illness or deaths. The difficulties of determining true impacts of food or water attacks associated with political motives are partly the reason this inventory starts post–World War II.

Our search phrases were only in English, and we did not attempt to replicate the search in other languages. We focused mostly on deaths in our results, because death is easier to confirm and link to attacks than episodes of illness, especially when illness is relatively mild. This narrow emphasis makes it possible to more clearly compare impacts from different types of agent or opportunity, but it neglects the importance of disabling illness.

We excluded smaller and purely targeted events so that we could focus on those incidents that generate the greatest public health response. Our interest was also in poisoning via food or water and not by other means. These last 2 criteria meant that this study omits an exceptional poisoning incident in London, UK, in 2006,^51,52^ which generated an enormous public health response because of suspected widespread environmental exposure to unintended targets (while still lacking our eligibility criterion of indiscriminate exposure via ingested food or water).

We did not analyze attacks by time or space: Other inventories have undertaken geographic categorization^1^ or trend analysis.^6^ Any spatial or temporal categorization or even trend analysis for these types of events, given how they tend to be documented, is subject to variable biases depending on the public appetite for such news, government policy about managing such information, news reporting infrastructure in the locality of the attack, probability of events being described in English-language reports, changes in monitoring or detection methods, and information sharing habits among would-be perpetrators. Therefore, it becomes difficult to make the data meaningful with reference to time or space. Mohtadi and Murshid^6^ attempted to overcome the underlying data uncertainties using extreme value theory, concluding that such attacks are becoming larger and more frequent. They did not adjust for recent population growth. With regard to the contextual factors explored in this article, brief assessment suggests that the context of attacks (especially types of targets, motives, moment of opportunity) in high-income countries tended to be similar to attacks in low-income countries. One of the few intercountry differences we can observe is that attacks related to business rivalry are much more likely to be reported in China than in other countries.

## Conclusions

Although the past cannot predict the future, our inventory of past attacks suggests that most realized poisoning opportunities and deaths from such attacks are likely to be in the context of catering for groups by family or other fellow community members. In contrast, and without wishing to endorse complacency, this inventory of past attacks suggests that attacks are less successful when targeting manufacturers or retailers. We cannot exclude the possibility that commercial pressures have encouraged underreporting, but it is also likely that many manufacturers and retailers have embraced mostly effective practices to minimize the human health risks of deliberate malicious contamination. A further useful security precaution could be to implement vetting procedures and background checks for volunteers preparing food, especially for larger or high-risk target groups (eg, police, refugees, members of religious minorities).

The small number of fatal cases resulting from publicly threatened attacks on food or water is reassuring and supports common-practice reassurances to the public from authorities, when such threats are first revealed, to take reasonable precautions but not to be unduly alarmed, precisely because past evidence indicates a low success rate for such threats and plots.

It is problematic that a number of dangerous agents are widely available. Limiting access to hazardous agents is effective in reducing the number of realized attacks (and may have similar impacts on likelihood of threatened attacks). Otherwise, given that food and water attacks may well recur, swift and accurate identification of the toxic agent(s) should be facilitated (with resources that health professionals find easy to consult) to achieve both effective treatment and other harm reduction.

Scenarios to prepare public health responses to possible attacks via food or water should consider diverse plausible contexts in how the adulterated food or water is delivered (in the home, at community events, etc), as well as the likely available contaminants. Moreover, it seems probable that the motive for attack as well as the nature of the perpetrator (eg, government, individual, member of ideologically driven group) and their motive(s) will often influence the mode, true intent, magnitude, and agent of attack.

## Supplementary Material

Supplemental data

Supplemental data

Supplemental data
