# Supplementary material for: Contextual Factors Among Indiscriminate or Large Attacks on Food or Water Supplies, 1946-2015
Source: Health Secur. 2016 Feb 1;14(1):19–28. doi: 10.1089/hs.2015.0056 (PMC5076485; doi:10.1089/hs.2015.0056)
Supplement: Supplemental data [file Supp_S1.docx]

Searched in March to July of 2015, but many of these sources were only updated to a certain date, eg: mid 2013 for *START*. Each inventory document and the specific databases cited were both hand-searched for eligible incidents.

| Inventory document | Specific source databases that we also consulted |
| --- | --- |
|  |  |
| Carus 2002 ^1^ |  |
|  |  |
| Dalziel 2009 ^2^ | National Counter Terrorism Centre, *Worldwide Incidents Tracking System*  Terrorism Knowledge Base (MIPT), which became the National consortium for the Study of Terrorism and Responses to Terrorism (START) *Global Terrorism Database*  US Dept of State *Country Reports on Terrorism*  The National Poison Centre (Universiti Sains Malaysia) covers the period 1995-2009 and the Asiatic region  <https://github.com/gregdl/food_defence_incidents/blob/master/data/food.csv> |
|  |  |
| Gleick 2006^3^ | www.worldwater.org |
|  |  |
| Howitt 2003 ^4^ |  |
|  |  |
| Koukouliou et al 2009 ^5^ |  |
|  |  |
| Kroll 2006^6^ | http://hachhst.com/wp-content/uploads/2010/07/White-Paper_Water-as-a-weapon.pdf |
|  |  |
| Mohtadi and Murshid 2009 ^7^ | Pinkerton Global Intelligence Services  Weapons of Mass Destruction (Center for Nonproliferation Studies) |
|  |  |
| Motarjemi 2014 ^8^ |  |
|  |  |
| Purver 1995 ^9^ |  |

References

1. Carus WS. Working paper: bioterrorism and biocrimes: the illicit use of biological agents since 1900. Washington, DC: Center for Counterproliferation Research, National Defense University; 2002. https://fas.org/irp/threat/cbw/carus.pdf. Accessed January 26, 2016.

2. Dalziel GR. *Food Defence Incidents 1950–2008: A Chronology and Analysis of Incidents Involving the Malicious Contamination of the Food Supply Chain.* Singapore: S. Rajaratnam School of International Studies, Nanyang Tachnological University; 2009. http://www3.ntu.edu.sg/rsis/cens/publications/reports/RSIS_Food%20Defence_170209.pdf. Accessed January 26, 2016.

3. Gleick PH. Water and terrorism. *Water Policy* 2006;8:481-503.

4. Howitt AM, Pangi RL. *Countering Terrorism: Dimensions of Preparedness.* Cambridge, MA: MIT Press; 2003.

5. Koukouliou V, Ujevic M, Premstaller O. *Threats to Food and Water Chain Infrastructure.* Dordrecht, The Netherlands: ;Springer Science & Business Media; 2009.

6. Kroll DJ. *Securing Our Water Supply: Protecting a Vulnerable Resource.* Tulsa, OK: PennWell Books; 2006.

7. Mohtadi H, Murshid AP. Risk analysis of chemical, biological, or radionuclear threats: implications for food security. *Risk Anal* 2009;29:1317-1335.

8. Motarjemi Y. *Encyclopedia of Food Safety.* New York: Academic Press; 2014.

9. Purver RG. Chemical and biological terrorism: the threat according to the open literature: Canadian Security Intelligence Service; 1995.
