# Supplementary material for: Contextual Factors Among Indiscriminate or Large Attacks on Food or Water Supplies, 1946-2015
Source: Health Secur. 2016 Feb 1;14(1):19–28. doi: 10.1089/hs.2015.0056 (PMC5076485; doi:10.1089/hs.2015.0056)
Supplement: Supplemental data [file Supp_Data.zip › Supp_S2.docx]

Supplemental Data File S2: List of eligible poisoning events

**FOOD ATTACKS**

| **When** | **Where** | **Food(s)** | **Contaminant (dose)** | **Who did & why** | **Intended Target** | **Method** | **Impact (who affected & how)** | **Public Health Response and other outcomes or details** |
| --- | --- | --- | --- | --- | --- | --- | --- | --- |
| May 2015^1^ | Dongguan China | Tea Drink products (cartons) | Rat poison | Guan; ‘angry with the world’ | products @ 4 shops | Injected | 1 death & 3+ to hospital | Man arrested and confessed. All items confiscated from shelves; The public security bureau, hospitals, municipal food & drug admin & other relevant depts. working together to closely monitor developments & keep public informed |
|  |  |  |  |  |  |  |  |  |
| 2015 ^2^ | Afghanistan | Beans | ? | ? Taliban? | School kids | ? | 100 to hospital | Not clear. |
|  |  |  |  |  |  |  |  |  |
| 11 Jan 2015^3-5^ | Mozambique | Beer | ?; crocodile bile acused but not dangerous; probably pesticide, or malathion | Graciano Antonio; motive unclear | ? | ? | 72 died, 169+ more also ill | Those who drank the brew only in the morning had no signs of illness, [the Associated Press reported](http://abcnews.go.com/International/wireStory/52-people-die-drinking-poisoned-beer-mozambique-28146693) Monday. But those who drank in the afternoon were sick by the next morning. Woman who brewed the beer among dead. Suspicious objects found in barrel, samples to national lab. |
|  |  |  |  |  |  |  |  |  |
| 2014-15 ^6-9^ | NZ | Infant formula. **Threat only.** | Pesticide 1080; sodium fluoroacetate | ?anti-pesticide protestors but also maybe financial; 60yo man arrested Oct. 2015 | Public (babies) | Fonterra producer & Fed Farmers HQ | Only Threat, extortion plan | Well documented, Police & Food security response, Ministry of Health is mentioned. Mention of 40k samples tested, increased security at supermarkets and at manuf. plant. ppl are named we could contact |
|  |  |  |  |  |  |  |  |  |
| 11 Dec 2014^10^ | Zheijiang, China | Snacks | Rat poison | ? | ? | Beautifully packaged on child tricycle | 2 died, 2 more ill | Investigation ongoing and disputed |
|  |  |  |  |  |  |  |  |  |
| Aug 2014 ^11,12^ | Care home for elderly W. Bromwich UK | Water and juice drinks | bleach | Melissa Swift (worker); malice? | Colleague & residents @ care home | injected | 4 hospitalised, 28 others ill; all fully recovered | She made statements about intending to kill someone, confessed after questioning. No record of anti-social behaviour. |
|  |  |  |  |  |  |  |  |  |
| Apr 2014 ^13^ | Northern Nigeria | Group meal | Rat poison | Wasila(t) Tasi’u | Husband = Umar Sani | In meal | Husband + 3 others died | Some dispute, evidence relied on 7yo witness, could it have been accidental?, but was convicted |
|  |  |  |  |  |  |  |  |  |
| 19 Mar 2014^14^ | Shangpinglong, Yunnan, China | Snacks in bags = pizza rolls | Tetramine, rat poison | Zhao Jianzhi disgruntled employee | Kids | Left in room of napping kids | 2 died + 30 more ill | Not clearly described |
|  |  |  |  |  |  |  |  |  |
| 2013-2014^15^ | Gunma, Japan | Snacks & frozen fast food | ? malathion found | Toshiki Abe, employee | Aqli food snack co. |  | ~2843 ill, baby hospitalised | Product recall, police investigation, criminal proceedings may be ongoing |
|  |  |  |  |  |  |  |  |  |
| 24.12 2013 ^16^ | Greece | Coca-cola | Hydrochloric acid | Anarchist group | CC (& Nestle) | ? | Threat only | Items were recalled from shelves; Perps stated they didn’t want to harm people just the companies; none found in products |
|  |  |  |  |  |  |  |  |  |
| Dec? 2013 ^17^ | Loudi, Hunan, China | drink | Rat poison & herbicide | 34yo woman, MH probs | children | injected | 19 ill, 3 seriously, all recovered (?) | Arrested…. Mentally ill, not finding more details |
|  |  |  |  |  |  |  |  |  |
| 2012-2013 ^18^ | Afghn | Food? | ? | 6 attacks described & not claimed; +Taliban claimed 3 x atks with deaths | Police officers & security staff | ? | ~490 ill  19+ killed | “…unidentified substance at a police compound…, sources suspect that the event is part of a string of poison attacks targeting police officers in Afghanistan.” All but 1 deaths happened in incidents (claimed by Taliban &) where officers are poisoned & then shot |
|  |  |  |  |  |  |  |  |  |
| 2013 ^19^ | ? | Soft drinks | Mineral acid | ?; extortion possibly not for money | ? | Bottle contents replaced & sent to manuf. | Threat only | Not stated; no one ingested |
|  |  |  |  |  |  |  |  |  |
| 24 July 2013 | Kala Khel, Zabul, Afghnsn | Food | ? | Taliban claimed | Police officers | See ->; not included in above events | ? some ill | “The assailants first poisoned the officers' food, then abducted 12 of them. The outcome of the kidnapping is unknown. Qari Yousuf Ahmadi, spokesperson for the Taliban, claimed responsibility for the incident, stating that they intended to punish the abducted police officers because the officers had allegedly tortured local residents.” |
|  |  |  |  |  |  |  |  |  |
| May 2013 ^20-22^ | Hebei, China | Yoghurt drink | Rat poison = tetramine | rival kindergarten staff = Shi Haixia & Yang Wenming | ?kids | ? injected | 2 died | Rushed to hospital, investigation launched…, man and woman detailed & confessed, more details will be in Chinese |
|  |  |  |  |  |  |  |  |  |
| April 2013 ^23^ | Steinfeld, DE | Sandwiches | Rat poison | ? maybe not clear deliberate? | Car parts Factory workers | ? | 25 to hospital | Emergency services called; and MD said “that he’d look into protective measures, perhaps including fencing off the premises” |
|  |  |  |  |  |  |  |  |  |
| 2012^24^ | Torkham border post Afghanistan | Fruit, coffee, cooked food? | ? bleach? | Talilban claimed deaths that were denied | NATO troops = ISAF | ? | Traces of bleach detected before consumed | “Soldiers are now eating pre-prepared rations, and no one was affected. There is a full investigation that is narrowing down who was responsible," said Maj. David Eastburn, an ISAF spokesman. (BBC report too) |
|  |  |  |  |  |  |  |  |  |
| 29 Nov 2011^25^ | Toronto CA | ? restaurant | Rat poison or E. Coli | Animal Liberation of USA/  Canada | Chinatown restrnts | ? | ?threat only? | Hate crimes unit of police called in; no further info found |
|  |  |  |  |  |  |  |  |  |
| Oct 2011^26-28^ | Ra’anana, Israel | Grapefruit juice, House tap water and household food also laced | pesticides | Adnan Othnan (Othman Nassar) Nasaara; revenge x Jews | Jews | ? | 6 ill, of which 4 hospitalised, 1 in intensive care. including police officer investigating the break-in | <- Other suspects named as Atman Nasrallah and Hassab Addel Rachim: expected to face charges of attempted murder, breaking and entering, theft and conspiracy to commit a crime |
|  |  |  |  |  |  |  |  |  |
| 2011 ^29^ | Pingliang, Gansu China | Cow milk | Nitrite | ? ppl detailed | ? kids? | ? | 3 kids died, 36 others hospitalised | No further info; was deemed deliberate |
|  |  |  |  |  |  |  |  |  |
| 2010?-2011^30^ | Haizhu, Guangzhou, China | Rice, fish, mushrooms, noodles | Broamadiolone, chlrophacinone (rat poisons) | Zhang; extortion | supermarket | Poisoned | Threat and plot only | Poisoned foods removed from shelves; 2 local hospitals put on alert (slow acting poisons). Zhang Captured. No reports anyone ingested. |
|  |  |  |  |  |  |  |  |  |
| 2010^31^ | Gweru, Zimbabwe | Delicatessen items, supermarket | ? | Employees sabotaging each other, ‘Nice Time’ market | Each other/customers | ? | 20 hospitalised | Alleged by supermarket director = Chrispen Thomu that employees were deliberately sabotaging each other, no other evidence to substantiate or response recorded; Very possible boss was deflecting own responsibility for negligence. Market closed down. |
|  |  |  |  |  |  |  |  |  |
| 2010 ^32^ | USA | Salad bars & buffets (public restaurant) | Ricin or cyanide | Islamist terrorists (AlQ affiltd) | US public | ? sprinkle | Plot only | Public health bodies & restaurants put on alert not too dismiss any incident as food poisoning, other actions unclear |
|  |  |  |  |  |  |  |  |  |
| Aug 2009 ^33-35^ | Harvard Uni | Coffee | Sodium azide | ? | Colleague? | ? | Mild symptoms in 6 people (2 fainted, heart palpitations) | Security measures installed afterwards; not proven as deliberate but no plausible alternative |
|  |  |  |  |  |  |  |  |  |
| ?June 2009 ^36,37^ | Muge, Guangxi Zhuang China | ? (meal) | Rat poison | Li Shengguang, unclear dispute | Lu Zhikun, Cook at school | ? | 21 toddlers ill, of which 2 died. Cook unhurt | Was arrested in June 2009. Confessed. Put to death 29 May 2011. |
|  |  |  |  |  |  |  |  |  |
| 26.1. 2009 ^38^ | West London, UK | Curry | Aconite ferox | Spurned lover = Kaur Si ngh | Man, ex bf | Laced | He died, his gf recovered | “The rapid and fatal effect of the poison alarmed the hospital and police evacuated Mr Cheema's and Singh's homes, suspecting there may have been an airborne or chemical threat.” |
|  |  |  |  |  |  |  |  |  |
| 11 & 30 Aug 2009 ^39,40^ | Lenexa, Kansas | Salsa | Pesticide, methomyl | Disgruntled Employees= Yini De laTorre & Arnoldo Bazan | Customers | Contaminated | 40+ ill, some hospital visits | Police & FDA investigated, Epidemiology & Public Health bodies involved, emergency closures & loss income to restaurant = Mi Ranchito; huge details on kdheks reference |
|  |  |  |  |  |  |  |  |  |
| April - July 2008 ^41^ | NY NY, USA | Gerber baby food; intent to scare shown by repeat videos | Cyanide or rat poison; hoax only to get ppl to his porn videos? | Anton Dunn (black himself); attn. seeking | Babies, esp. black or Hispanic | ? | Threat only, parents frightened on Internet | FDA looked for tampering, so did Gerber, imprisoned for 1 yr: http://www.nydailynews.com/news/crime/man-behind-youtube-hoax-gerber-baby-food-poisoning-threat-joke-article-1.370559 |
|  |  |  |  |  |  |  |  |  |
| April 2008 ^42^, arrested Nov ‘08 | Germany (Bonn, Cologne, Dusseldorf) | Gherkins | Cleaning fluid (detergent) | 67 or 73yo pensioner (money), lived in Bonn =  Z Horst; extortion | Lidl supermarket | Poured in | One contaminated jar easily found | Letters to newspapers & Lidl headquarters, “detailing his threats and demanding a six-figure sum of money”; described as 2^nd^ such blackmailer in 6 months, article says “no longer any danger to the public” would need to search in German to find more; easily apprehended when he tried to meet someone for blackmail money; has privacy under German law; |
|  |  |  |  |  |  |  |  |  |
| Oct 2008 ^43^ | Koln, DE | ? | ? | 44yo entrepeneur; extortion | Lidl | Vague threat | Threat only | JN says no products pulled from shelves or tested, just police contacts |
|  |  |  |  |  |  |  |  |  |
| Sept 2008 ^44,45^ | Nuristan Afghanistan, NATO base | Bread? | poison | Taliban claimed they did, Ajab Khan | Military | One report said cook poured bleach in | 300 ill, perhaps some deaths | Reports on this incident are highly inconsistent, NATO insisting it was not deliberate malicious event, Taliban taking credit, no followup but is in US State dept. as a terrorist incident. Everyone agrees that many were ill. |
|  |  |  |  |  |  |  |  |  |
| ?Aug 2008 ^46^ | Business ppl conference, Sweden | Cafeteria food | Shigella dysenteriae | claimed by left-wing extremists; ?motive | ? | ?? | 140 ill, 5 admitted to hospital | Cafeteria closed and investigated, nothing found there. Sweden’s security service investigated and reached English language press after claim by political group. Followup info inadequate. |
|  |  |  |  |  |  |  |  |  |
| 20.8. 2008 ^47^ | Vogelsberg, Hesse, DE | ? misc | ? “foul smelling substance” | 65yo pensioner (money), lived in V’g; extortion | Lidl | ? contamination | Threat only | Perhaps none, JN found that no items were removed from shelves only police contacted. Culprit *has privacy under German law* |
|  |  |  |  |  |  |  |  |  |
| 2008 ^48,49^ | Haikou, Hainan | Rice | Rat poison | Huang Yuchen & Wu Shushun | Rival business | ? | 3 died + 9 in hospital | Arrested… |
|  |  |  |  |  |  |  |  |  |
| 2008 ^50^ | Hong Kong | Cakes | psychotropic | Jannifer Chan Mei-fung | ? | ? | 6 ill + 2 hospitalised | Convicted; Judge Joseph Yau described Chan as "a dangerous character" who not only suffered from paranoia but was also manipulative and cunning and in desperate need of treatment. |
|  |  |  |  |  |  |  |  |  |
| March 2008^51^ | Israel | Restaurant | ? white and tasteless | Illegal Arab workers = Ihab Yehye Ahmed Abu Riyal & Anas Mustafa Said Salum | Customer | Slipped | Plot only | “arrested on March 19 in a joint operation by intelligence officers from the Israel Security Agency (Shin Bet) and the Israel Police, several days before the attack - which would have been the first of its kind - was to have been carried out.” |
|  |  |  |  |  |  |  |  |  |
| Feb 2008 ^52,53^ | Longgang, Shenzhen China | Hot lunch foods, possibly also water | Sodium nitrite | Business rivals | Business rivals | Spread on food stalls local market | 63 ill, including 2 died | Epide. & criminal investigation, since they say why it happened, presumably caught & convicted; main source is in Chinese |
|  |  |  |  |  |  |  |  |  |
| Jan 2008 ^49,54^ | Tokyo, Japan | Bottled green tea | Glyphosate | ? | ? | ? | None reported | Bottles were evidently tampered with, others in same batch not contaminated; could dispute if meant to harm? May be Japnse lang article |
|  |  |  |  |  |  |  |  |  |
| Jan 2008 ^55^ | Malaba, Tororo, Uganda | Maize & beans | ‘poison’ | Kalenjin tribesman, ethnic conflict | Kikuyu refugees from Kenya | Mixing | Caught in the act both times;  Plot only | 2 incidents at refugee centres, minimal info, 1 x beans poisoner at church & 2 x maize poisoners at school arrested, bean poisoner escaped, others? |
|  |  |  |  |  |  |  |  |  |
| 22 Jan 2008 ^56^ | Baghdad, Iraq | Cakes | Thallium | ?political | Air Force members | ? | 4 kids died; 6 others very ill | Epid. Investigation initiated 8 Feb 08 to link illness to cake |
|  |  |  |  |  |  |  |  |  |
| Jan 2008 ^57-61^ | Japan | Gyoza dumplings (frozen import from China) | Pesticide = methamidophos | Lu Yueting, unhappy temp. employee at plant in China | His employer | Injected in dumpling or Lined inside of packaging; not in the dough/filling (?) | 10 people ill | Mass product recall in Japan. Caused international tensions and cooperation. 5+ yr investigation. Some details about who was rushed to hospital and timing of illness & recognition of incident. Jailed for life in 2014. |
|  |  |  |  |  |  |  |  |  |
| 11 Oct 2007 ^62^ | Xiantao, Hubei | Breakfast buffet | Rat poison | Hu | Colleague(?) | ‘slipped’ | 1 died + 11 in hospital | Hu committed suicide, she was tax inspector with known resentments of colleagues |
|  |  |  |  |  |  |  |  |  |
| Aug 2007^63-65^ | Kings Lynn, Norfolk UK | Baby food | Bleach | Shane Ward; extortion | Morrisons Spmkt | Put | Threat and plot only | Risk assessments done.  Reference to multi-agency /partners response  Ward put bleach in a single jar of baby food in a jiffy bag in the vegetable section of Morrisons store in King's Lynn, Norfolk. Traced via his mobile phone registration. Massive recall of baby food jars at other stores, bomb disposal unit mobilised. Cost to store = £20k +. |
|  |  |  |  |  |  |  |  |  |
| 2007 ^19^ | ? possibly UK | Pastries | Peanuts (nut free products) | ? | ? | Piles of peanuts found on premises | No one ingested  ; Lost 5% of sales according to some sources | “closed for a week long deep clean to re-establish its nut-free status.” This incident is widely reported but never with specific details such as date or location. Read like an urban myth. |
|  |  |  |  |  |  |  |  |  |
| May-July 2007 ^66,67^ | Britain | Yogurt | Caustic soda | Philip McHugh, money extortion | ? | Put | Threat only | Caught, pleaded guilty, sentenced January 2008 |
|  |  |  |  |  |  |  |  |  |
| 2007^49,68^ | Charleston USA | Ground beef | Rat pellets | Karen L. Wyndham, disgruntled employee | Revenge on her boss | ? | Plot only, found before eaten | Arrested, confessed, convicted. |
|  |  |  |  |  |  |  |  |  |
| April 2007 ^49,69^ | Harbin, Heilongjiang | Porridge | Rat poison in water used to make porridge | ? | ? | In boiler water used to make porridge | 203 ill + 1 death | Heavily investigated, some dispute if deliberate |
|  |  |  |  |  |  |  |  |  |
| March 2007 ^49,69^ | Xixi, Yongkang | Stuffed buns | Rat poison | ? | ? | ? | 57 ill, no deaths | Heavily investigated, police believe was deliberate, Some dispute if this was deliberate |
|  |  |  |  |  |  |  |  |  |
| Dec 2003-Mar 2007 ^70^ | Yaroslavl, Russia | Coffee, caviar, others | Thallium | Vyacheslav Solovyov | Family | Various | 6 deaths | Confessed to all killings, including one that was unintended (daughter), after being detained in May 2007; died Dec 2008 |
|  |  |  |  |  |  |  |  |  |
| Aug 2006 ^49,71^ | Kigali City Rwanda | Bucket of beer | Rat poison | Savera Nyirashongole (?paid) | ? | ? | 1 ill, many critical, 57 hospitalised | SN was arrested, said she was paid to poison people, followup unclear |
|  |  |  |  |  |  |  |  |  |
| Jul 2006 ^49,72,73^ | South Korea | Coca Cola | Herbicide | Woman=Park; unclear | Big company | injected | 1 x Man hospitalised, | Product recall off shelves, suspect arrested, items soon back on shelves in new packaging, 2 other bottles had the contaminant |
|  |  |  |  |  |  |  |  |  |
| May 2006 ^49,74,75^ | Papua New Guinea | ? | Cyanide | Local people | Relatives | ? | 2 died | Allegations this was widespread, but not well documented, hospital said some reports of incidents were untrue |
|  |  |  |  |  |  |  |  |  |
| Jan- May ’06 ^76^ | Hamburg, DE | HCl & botox | 23% HCl -> babyfood/marmelade , and wine with botox | Male merchant age 48; extortion | ? | (mix) | None found in food, no one ill | Babyfoods were removed from shelves (none found to be contaminated), public warned on mouthwash or babyfood with marks on lids, payment was made in sting operation (guy caught) |
|  |  |  |  |  |  |  |  |  |
| 2003-2006 ^77-80^& beyond? | Italy, at least 3 cities, more often north IT | Fruit juices, probably soft drinks too | Various, herbicide, fungicide, unclear, | Unclear, Mix of original & copycats, maybe all copycats | ? | ? many were injected | 5+ ill | Grouped together, perhaps incorrectly, but rarely bleach, and less often ingested, came in an extended wave from products bought in huge range of locations after initial Acquabomber events. Toddler (Shaden) suggested food attacks ^81,82^ **excluded** because of lack of physical evidence, unlikely coincidence [& police suspicions](http://www.disabiliforum.com/forum/2699-bambina-intossicata-sospetti-sulla-madre.html). Shaden maybe taken into care? |
|  |  |  |  |  |  |  |  |  |
| (2005 &) Feb 2006 ^83^ | CT, USA | (prune &) Grape Juice | Dishwashing liquid | Wendell Woodroffe, revenge to boss | Church goers | Spiked | 44 ill (+1 prune?) | Found guilty, Given probation |
|  |  |  |  |  |  |  |  |  |
| 2005^84^ | Dallas TX | Pastries | Feces | Behrouz Nahid Mobarekeh; ? mentally ill & had complaints against the store | ? | Sprinkled dried or grated feces | ? | Caught on tape, arrested, deemed to not be politically motivated, clear that it was deliberate, but whether he meant to harm could be unclear. Went to jail but not clear for what crime, defense did good job showing the dried feces couldn’t actually harm anyone |
|  |  |  |  |  |  |  |  |  |
| Oct 2005 ^85^ | Australia | Fruit concentrate | ? | ?; extortion | Company = Berri | Contaminate | ? | Stopped production at South Australia plant, at least for a while. |
|  |  |  |  |  |  |  |  |  |
| Sep 2005 ^49,86^ | Sanhe, Hebei, China | Porridge/meal | Dushuqiang (rat poison) | ? | ? | ? | 23 hospitalised, 30 more ill | Police believed it was deliberate, further reports not available |
|  |  |  |  |  |  |  |  |  |
| 2005 ^87^ | West Bank (Palestine) | livestock | Rat poison or other chemicals | Israeli settlers; political | West Bank Palestinanas | rat poison (2005) | None apparent | No apparent PH response yet, but threat to food security; Amnesty Intnl may have a full report which describes a PH response |
|  |  |  |  |  |  |  |  |  |
| 2005 ^47^ | Lidl Germany | “foods” | salt and denatured alcohol | ? man; extortion | Lidl | In “foods” | ?was this threat only? | ?. Culprit was imprisoned 4 y 9 m. Name unpublished because has privacy under German law |
|  |  |  |  |  |  |  |  |  |
| Jun-Jul 2005 ^88-91^ | Sydney, NSW, Aus | Candies (Mars & Snickers) | ? pest poison in bar sent in | ? not caught ; extortion | Star City Casino | ? | 19 ppl complained they felt ill | Threatening letters sent on 8 & 15 June + 1 July with one contaminated Snickers and 7 contaminated bars on shelves; Massive widespread product recall & 3 miln destroyed, plus publicity. Extortion. |
|  |  |  |  |  |  |  |  |  |
| May 2005 ^92^ | Taichung, Taiwan | Energy drinks | Cyanide | Wang Ching-chan; extortion to store chain | ? | ? | 1 died, 3 others to hospital | “He attached labels that said "I am poisonous" to the bottles, but several people who failed to notice or thought the labels were an advertisement drank the beverage”; live confession on tv, sentenced to death |
|  |  |  |  |  |  |  |  |  |
| April 2005 ^93^ | Jahra, Kuwait | Drink | Rat poison | Muayad Al Saeedi | All 8 his children+ self | Forced them to drink | 5 children died | He confessed, was let off by claiming poor mental health |
|  |  |  |  |  |  |  |  |  |
| 2004 & June 2005 ^94^ | Glasgow | McVite’s Biscuits | Weed killer, dead rat | John McCulloch (employee) ; extortion | United Biscuits managers | “put” | Threat only, none found | *“*The threat sparked a major search at the factory in Clydeford Street, Tollcross. Weedkiller was found but none of the products were affected.*”: community service for the perp* |
|  |  |  |  |  |  |  |  |  |
| 2004 ^95^ | France | Chocolates, cheeses, drinks | ? | AZF 2?; extortion for political aims | manuf/public | ? | ? threat only | ? ; other kinds of threats from this group are ongoing; believed to be Islamist |
|  |  |  |  |  |  |  |  |  |
| 2004 ^96^ | KA USA | Cake, rootbeer | Antifreeze, lye | Donna Ozuna-Trout & Ralph Trout | Neighbours (2 adults + 2 kids) | Sent contaminated food | Not quite Threat only | Was part of intimidation campaign against the Eickhoff adults, but cakes easily could have been eaten by the kids & friends. Trouts were found guilty |
|  |  |  |  |  |  |  |  |  |
| 2004 ^49^ | Vietnam | Rice | ? rodenticide | Vang Thi Dinh & Giang Dung Xu | Neighbours | Mixed with sticky rice | 5 affected | Inadequate detail |
|  |  |  |  |  |  |  |  |  |
| 28.6. 2004 ^97^ | Samut Prakan, Thailand | Chocolate drink | Lannate (pesticide) | Thananya Hommak, teazcher, money woes | Children | Mixed | Thananya & 28 children taken ill, none died | Reports about arrest & her money troubles, but followup unclearly documented; unreliable source says Thananya got 5 yr sentence. |
|  |  |  |  |  |  |  |  |  |
| May, June 2004 ^98^ | Irvine CA | Gerber baby food jars | Castor bean extract (almost ricin) | Unknown; related to some kind of extortion or threat | ? | ? | Babies ingested but no harm | “ricin was not in the purified form that can be deadly”… The jars of Gerber Banana Yogurt also contained notes that tried to blame an Irvine police officer as poisoner. FBI investigated. No arrests made. |
|  |  |  |  |  |  |  |  |  |
| April or May 2004 ^99^ | Afghanistan | Biscuits | ? | ?  ?Taliban | School girls | Handed to them | 3 girls ill in hospital | Apparently crime of convenience rather than specific target |
|  |  |  |  |  |  |  |  |  |
| 2004 ^49,100,101^ | Tongchuan Shaanxi, China | Scallion Pancakes | Rat poison, dushuqiang | Any rival business user (coal mine workers) | Rival business | ate | 68 of 74 victims went to hospital 55 employees of the mine and 14 students. No deaths?. | Tests made by provincial disease prevention, not clear what other prevention measures taken. Police & mine administration investigated |
|  |  |  |  |  |  |  |  |  |
| Dec 2003 ^102^ | Hechi college, Yizhou City china | Breakfast | Tetramethylenedisulfotetramine | ? | College students | ? | 76 ill, no death? | ? reported as deliberate, but disputable evidence for that. Police investigated. Lots more here: http://www.sciencedirect.com/science/article/pii/S0379073810003221 |
|  |  |  |  |  |  |  |  |  |
| Nov 2003 ^103^ | Hunan, China | Popcorn & citrus | Rat poison | Wei Entan, revenge on x-lover | Children | ? | 27 ill including 2 died (targets) | Caught by police |
|  |  |  |  |  |  |  |  |  |
| 23.9 2003 ^49,104,105^ | Primary school, Yueyang Hunan | Baking powder | dushuqiang | Xu Guohua, not clear why | ? kids | Mixed | 161-215 poisoned | Apparently put to death |
|  |  |  |  |  |  |  |  |  |
| April 2003 ^106-108^ | New Sweden Maine (several books on this) | Coffee | Arsenic | Daniel Bondeson; unclear, he committed suicide?? | Church goers | ? | 16 ill, 1 quick death, 1 death 3 yrs later | Nurse Rose Tanguay given huge credit for recognising the poison within 12 hrs later confirmed by lab tests, then only a nursing student, sole employee on duty at poison centre, antidotes flown in, still works there, good details in Bangor article |
|  |  |  |  |  |  |  |  |  |
| May 2003^109^ | Cologne (DE?) | ? | Potassium cyanide | Mentally ill man | ?may be same as below Czech threat | ? | Threat only | Police took threat seriously & arrested him. Still trying to find details; police = Zdenek Jágrová & Petra Čimová |
|  |  |  |  |  |  |  |  |  |
| 25 May-27 June 2003 ^110^ | Zhejiang, China | Beverages | Rat poison | Chen Fuzhao | Homeless people, dogs before that | Mixed | 16 died including an unintended social worker, plus another person who recovered | 500 police involved. Confessed and executed, said to be politically motivated but that is somewhat disputed. Attempted poisoning of school children also documented in related reports. |
|  |  |  |  |  |  |  |  |  |
| *May 2003 ^111-115^* | Czech Republic | Hospital food, Motol Teaching & General Teaching Hospitals | cyanide solution (potassium cyanide) | Extortionists= Patrik Jirsa & Petr Hirjak | Pts | ? | Only threat though vial of 3% P-cyanide was found on hospital premises & others sent to authorities | *Unstated, but definite, sentence to prison & compensation ordered to Health Ministry & the hospitals which tightened security (guards and food prep supervision procedures); high costs to the hospitals mentioned, food distributed in sealed packages. Extended blackmail threats & negotiations for a spell. Nestle, Coca cola & Pepsi threats mentioned here: http://zpravy.idnes.cz/muz-hrozil-coca-cole-otravenim-limonad-dyz-/krimi.aspx%3Fc%3DA030513_222432_krimi_pol&usg=ALkJrhiAv3v-6_Qiw_wwYibN6Lr2IdfmdQ* |
|  |  |  |  |  |  |  |  |  |
| 2003^105^ | Ningbo, Zheijiang, China | Soya & chilli sauce at breakfast buffet | Dushuqiang | Yan Wancai, possibly business dispute | Restaurant owner/patrons | Put | 60+ made ill | Sentenced to life in prison |
|  |  |  |  |  |  |  |  |  |
| 2003^49^ | Minzhu, Sichuan  China | Mutton soup | Dushuqiang | Zhong Yongjin, family dispute | Wife & her relatives | Put | 5 died | He committed suicide soon after |
|  |  |  |  |  |  |  |  |  |
| 2003 ^49^ | Hubei, China | Meal, rice at funeral | Rat poison | Chen Xiaomei, alleged multiple family disputes | Family | Added | 10 dead & 23 very ill but recovered | No followup to say she confessed (widow of funeral man) |
|  |  |  |  |  |  |  |  |  |
| 23.1 2003^116,117^ | Hong Kong | Rice +? | Rat poison | Huang Zhuandi, to frighten cheating husband | Leung Kamshing (husband) | Slipped | Leung & co-worker Liu Qunying -> intensive care | Huang pleaded guilty; very detailed report available on clinical presentation, but says almost nothing about PH response. |
| 2002-03 ^118^ | Britain | Food | Ricin | Islamic militants | Military base staff | Lace | Plot only, if that much | Ricin was found in appt. of 6 arrested men, and investigators speculated this is why they had it (all tenuous). |
| Dec’02-Jan’03 ^104,119-121^ | Michigan USA | Beef | Nicotine (pesticide) | Supermarket employee = Randy Jay Bertram, 39yo; disgruntled | Wanted to get boss into trouble | Poured a bottle on prior to packaging, 31 Dec | 92+ ill, at least one hospitalised | Quite extensive investigation, supermarket recalled, many tests, tracing, etc. Lots of news coverage if search for his name. Got 9 yr prison sentence & paid $12k in restitution. Pled Guilty. His intention wasn’t to hurt people, but his indiscriminate actions could have hurt someone so keep? |
|  |  |  |  |  |  |  |  |  |
| *24 Nov 2002 ^102,122^* | Huangpo, Guangdong, China | Corn Porridge | Tetramethylenedisulfotetramine | Huang Hu; business rival | Rival nursery kids & staff | Added to table salt | 70 kids ill, 2 adults ill, no deaths | Many to hospital, ?unclear, : executed 3.1.2003 http://www.nytimes.com/2003/11/17/world/rat-poison-murder-weapon-of-choice-in-rural-china.html |
|  |  |  |  |  |  |  |  |  |
| Nov 2002 ^49,123^ | Changde, China | Breakfast | Rat poison | He Zhaohai, Tang Bixin, pay dispute | ? | Put | 193 made ill | Arrested, mentions a 14yo in Changde who poisoned restaurant food but few details available |
|  |  |  |  |  |  |  |  |  |
| 10.11 2002 ^124^ | Anqing, Anhui, China | (food) | Tetramine | Jiang Xingyou (bus. Rival) | Rival eatery customer | adulturated | 2 deaths, unknown ill | Apparently there were several incidents where he poisoned their food |
|  |  |  |  |  |  |  |  |  |
| Sept 2002^125,126^ | Jerusalem Israel | Food & drink | ? | 3 Arab men working in Israel (?political) | Customer | ? | Plot only | Lots of soul searching after arrests, panics about whether Arab employees were safe to have. Sentenced = Kianyah & ? |
|  |  |  |  |  |  |  |  |  |
| Sept 2002 ^102,104,127,128^ | Nanjing, China | Bakery/snack shop products | Tetramethylenedisulfotetramine | Chen Zhengping (see murderpedia) | Rival business | ?somehow added | 400 ill, 38 or 42+ died, 100+ to hospital | Shop was closed, owner taken into custody, 10 hospitals involved & 500 medical staff mobilised to attend pts. People collapsed outside the shop, massive event, CZ confessed & sentenced to death |
|  |  |  |  |  |  |  |  |  |
| Autumn 2002 ^129^ | Sangamon USA | Food and drink | Thallium | Adetokundo Fayemi, boyf. | Alice Minter (girlf) | ? | Alice long term problems, 7 others poisoned | Convicted in 2006 |
|  |  |  |  |  |  |  |  |  |
| 26.9 2002 ^124^ | Xi’an, China | Restaurant food | Tetramine | “farm girl” pay dispute | Her boss | Poisoned | 5 died | ? |
|  |  |  |  |  |  |  |  |  |
| August 2002 ^124^ | Tongchuan, Shaanxi, China | Milk | Tetramine | Zhong Dongxiang, to marry mistress instead | His wife | ? | Wife dead + 15 others made ill | About 1 ton of milk discarded for fear of also being contaminated |
|  |  |  |  |  |  |  |  |  |
| August 2002 ^130^ | Osaka, Japan | Coffee sugar | potassium cyanide and sodium azide | Kazuhiro Meguro; unhappy at work | ? colleague | Mixed | 3 ill including 1 in hospital, 2 more spat out | Police investigated |
|  |  |  |  |  |  |  |  |  |
| 19.5. 2002 ^104^ | Nyazura, Zimbabwe | Tea | Pesticide | Johanne Marange Apostolic Church, a  Christian fundamentalist group, | Selves & ? | ? | 7 died and 47 ill | Deceased included a small child. |
|  |  |  |  |  |  |  |  |  |
| 30 Jan 2002 ^49,104,124^ | Linxiang, Hunan | School lunch (veg) | Rat poison | Angry student taking revenge on teacher | School children | scattered | 92 children ill | “Although the Miscreant intended to poison only the teacher, the tainted ingredients were unwittingly used by the kitchen staff and served throughout the school population” |
|  |  |  |  |  |  |  |  |  |
| 1999-2002 ^49,131^ | Utrecht | Yogurt, pudding, packaged desserts | Pesticides & rodenticide | Frans van Laarhoven; extortion | ? | Injected | 4 people ill | Found guilty, paid compensation to victims & companies |
|  |  |  |  |  |  |  |  |  |
| 27.9-2.10 2001 ^132^ | Hong Kong | Mashed potatoes & choc. Drink | Carbofuran | Ma Chiu-sing & Kwok Kuk-heung | Politicians (extortion) | ? | Threat only | Backed up threat to restaurants, demanding senior officials resign, with easy to find contaminated foods; Ma pleaded guilty |
|  |  |  |  |  |  |  |  |  |
| 8 Aug 2001 ^49,104^ | Ningxiang, China | Restaurant noodles | Rat poison | ? | ? | ? | 120 ppl in 16 restaurants ill | ? |
|  |  |  |  |  |  |  |  |  |
| 2001 ^49^ | Nanyang, China | Soft drinks | Rat poison | Du Keping, practice | ? | Spiked at supermarket | 1 died, 4 very ill, ~12 more ill | Was practicing before he poisoned his son & wife |
|  |  |  |  |  |  |  |  |  |
| 1998-Aug 2001 ^133^ | Binh duong, Vietnam | Water or food | Cyanide | Le Thanh Van + boyf; money & spite | Family, people to steal from | ? | At least 13 death & unknown ill | Arrested & sentenced to death. Fake wills were produced after their deaths leaving money to her. |
|  |  |  |  |  |  |  |  |  |
| 1998 & 2001^134^ | NSW, Australia | Various products | ? | Richard Lenton Hennell; extortion | Sanitarium Health Foods Co. | ? | Threat only | Prompted mass expensive recalls in 1998 & 2001; no contamination ever found & not clear if vandalism or malice threatened; demanded money. |
|  |  |  |  |  |  |  |  |  |
| July 2001 ^135^ | NSW, Aus | ? | “poison” | Sef Gonzales; possible psychopath | Family | ? | Threatening letters only, no record of implementation | Did really poison his mother & stab whole family after that |
|  |  |  |  |  |  |  |  |  |
| Jan 2001 ^49,136^ | Jinan, China | Rice porridge | Du Shu Quiang | Li Feng, grudges | Classmates | ‘put’ | 2 died, 7 others ill | ?; executed in September 2002 |
|  |  |  |  |  |  |  |  |  |
| 28.6 2000 ^137^ | Hamamatsu, Japan | Curry | Insecticide | Extortionist | Ezaki Glico Co. | Laced | Threat only | Obviously tampered box on shelf + extortion threats for money, not clear if ever caught |
|  |  |  |  |  |  |  |  |  |
| 1998-May 2000 ^138^ | Israel | Eggs | Sabotage to certification system | Palestinians; poltical | Israelis? | Used counterfeit stamps on expired & salmonella ridden eggs | Unclear ? 2 deaths from salmonella illness after eating eggs during this period | Steps taken to better check the stamps; deception went on for 18 months before discovery; more original sources are unavailable |
|  |  |  |  |  |  |  |  |  |
| May 2000 ^139,140^ | Laval Uni, Quebec | Coffee | Arsenic | ? animal rights activists suspected | ? | Laced | 20-30 ill | “After making some inquiries, she and others realized that one common trait the ailing co-workers shared was that they all had been drinking the same type of coffee blend, purchased from the machine on their floor.  Authorities have seized the machine. Inside, one of the blends of grounded coffee had been sprinkled with some white grains, said Lieutenant André Filion, a spokesman for the police of suburban Ste-Foy, where Laval is located.  Test results completed Wednesday identified the white residues as a form of arsenic.  Jocelyne Dagenais, a spokeswoman for the provincial Agriculture Department, said the quantity of the poison found in the machine "was not accidental." |
|  |  |  |  |  |  |  |  |  |
| 17.3. 2000 ^104^ | Uganda | ? | ? | ?; religious extremists | ? | ? | Hundreds poisoned | Cult mass suicided & burnt selves up; dozens of bodies found afterwards deemed to have been murdered, but means unclear |
|  |  |  |  |  |  |  |  |  |
| 8 /3 2000 ^104^ | Jalaludin Afghanistan | School food | ? | ?; political? | ? school kids | ? | 2 died, 60 others unconscious | ? evidence is disputed whether deliberate or hysteria, only Mohtadi & M. and Pate et al 2000 mention deaths |
|  |  |  |  |  |  |  |  |  |
| 2000 ^104,141^ | Palestine | Agric. Fields | Sewage water | Israeli settlers; political | Palestinians; ? to make them leave | Sewage flooded onto land | ? | Only disruption to agricultural plans and livestock deaths(?) Plenty stories about deliberate contamination of wells by extremist Jewish settlers, but no reliable source to cite for wells, and no PH response detailed, either |
|  |  |  |  |  |  |  |  |  |
| 2000 ^49^ | Qingfeng, Henan | Food+water | Rat poison | Wang Pengjiao, jealous | Neighbours | Put | 3 dead, 2 more ill | Minimal details, was executed |
|  |  |  |  |  |  |  |  |  |
| 2000 ^49,124^ | Jiangxi, Nanchang | Meal | Rat poison | Migrant worker, why? | Others in boarding house | Laced | 48 ill | Very limited info |
|  |  |  |  |  |  |  |  |  |
| Aug 1999 ^142,143^ | Maryland USA | Fruit juice | ‘poison’ = drain cleaner(?) | Lorraine B. Gray, personality disorder | Coworkers at a charity | Gave bottles out | unclear actual effects | Guilty of 31 counts of assault |
|  |  |  |  |  |  |  |  |  |
| 1999 ^144^ | USA | Milk, specific plant | Biological agent | ? ; extortion | ? | ? | threat only | Sequestered alleged milk for 12 hrs but found to be clear of pathogen. State agric. Dept with local law enforcement and public health organisations; FBI opened a criminal investigation |
|  |  |  |  |  |  |  |  |  |
| March 1999 ^104^ | Luoyang City, China | Restaurant food | Nitric acid | Business rival | ? | ? | 148 ill | ? |
|  |  |  |  |  |  |  |  |  |
| March 1999^85^ | NSW, Austr. | Coca Cola | Glass, poison | ?; extortion | Coca Cola Company | Contaminate | Threat only | ? |
|  |  |  |  |  |  |  |  |  |
| 1999^49^ | Arizona | Soft drinks | Risperdal,, anti-psychotic | Daniel Arnulfo Ortega Jr. | Teacher + pupils | ‘lacing’ | Presumably very ill | (only in Dalziel): DAO charged with attempted murder after teacher drank soda; 2 students also drank but uninjured. |
|  |  |  |  |  |  |  |  |  |
| Dec 1998 – Jan 1999 ^145,146^ | Fresno USA | Salt in Restaurant dishes | Methomyl (licensed pesticide) | ?; possible employee-owner dispute | ? | ? | 107+ ill, dozens went to hospital, no deaths | Restaurant closed for almost 1 month, interviews with all staff, check of all ingredients found the salt mixed with pesticide, Feds, state officials & CDC investigated. Salt producer not implicated. |
|  |  |  |  |  |  |  |  |  |
| Dec 1998 ^144^ | USA | Meat | HIV | ?; extortion | Co. = Emmpak Foods | Contaminated | Threat only | USDA searched the plant; WI dept. of health, FBI & police investigated supported by CDC & OSHA; 800 ppl interviewed |
|  |  |  |  |  |  |  |  |  |
| Nov 1998^147^ | Brown Uni NH, USA | Curry: veg + chicken | Iodine-125 (radioactive), stolen from Uni lab | Cheng Gu; jilted lover, could be no harm meant | Yuanyuan Xiao (ex gf) | Gu cooked & gave her | Xiao & flatmate ingested, unhurt | Discovered when Xiao set off a Geiger counter at a Uni lab. Led to investigation of Xiao & then her flat, the tainted food found there. Flat-mate unintended victim. Dose too low to cause harm. |
|  |  |  |  |  |  |  |  |  |
| 1998^49^ | USA | Salt and pepper, coffee | Iodine pills | Daughter, ? | Mother, | Put | Mother & friend ingested | Girl shot her mom a year later |
|  |  |  |  |  |  |  |  |  |
| 1998^49^ | Japan | Soft drinks | Insecticide | School girl | 27 classmates + teacher | ? poured & replaced? Posted to them | Only one kid drank, he went to hospital | Most could tell it smelled wrong |
|  |  |  |  |  |  |  |  |  |
| April 1998^49,148^ | Villavicencio Colombia | Wine | Cyanide, ammonia & methanol also found | ? | Priests and church volunteers | Laced, sent as anon. Easter gifts | 2 deaths, 2+ others went to hospital | 24+ parish priests received the poisoned wine and sweets+biscuits parcel gifts. Warnings went out after the initial deaths which prevented others drinking the tainted wine. Was blamed on satanic cultists by a bishop. No report of arrests. |
|  |  |  |  |  |  |  |  |  |
| 1998^49^ | China | Sweets | Rat poison | Dao Ruiying; revenge to parents | Neighbours’ children | ?laced | 5 children died | ?. She was executed; motive of revenge against the parents is mentioned in another description of AP article that Dalziel cites. |
|  |  |  |  |  |  |  |  |  |
| 1996-1998 ^149^ | Yongfeng, Zheijiang | ? | Rat poison | Jealous farmer | Neighbours | ? | 4 ppl dead, others ill, 12 buffalo poisoned | ? |
|  |  |  |  |  |  |  |  |  |
| July 1998 ^104,150-154^ | Wakayama, Japan | Curry | Arsenic & cyanide | Masumi Hayashi, wanted money insurance policies | Specific indiv. But got loads others | Poisoned curry at a festival, maybe also friends at dinner parties | 2 adults + 2 kids died, 60 (63?) ill | Japanese description makes PH response sound brief; inspired copycats. http://jikenshi.web.fc2.com/newpage252.htm |
|  |  |  |  |  |  |  |  |  |
| Dec97- Jan ’98 ^49^ | German cities | Jam | Rat poison | ? (unavail); extortion | Supermarket custom | Spiked | ? | Culprit was caught and confessed, never much info available from Germany |
|  |  |  |  |  |  |  |  |  |
| May 1997^49,85^ | Sydney Australia | Nestle products= yogurt & coffee | Poison | ‘former policeman’= Mark James Milton; extortion | Sydney supermarkets | Inject | ?threat only | ? |
|  |  |  |  |  |  |  |  |  |
| April 1997^49,155^ | NYC, NY USA | Cookies | Rat poison | Hyung-Oh Cha; extortion | Supermarket customers | Laced | One person consumed & was ill & recovered | He pled guilty, news report of May 1997 |
|  |  |  |  |  |  |  |  |  |
| April 1997 ^49^ | Germany | Mustard, milk drinks, more? | Cyanide, acid, ? | ? (unavail); extortion | Thomy, subsidiary of Nestle | Spiked | ? | Sentenced to 11 years in September 1999 (more info maybe in German) |
|  |  |  |  |  |  |  |  |  |
| Feb 1997 ^156,157^ | NSW, Queensland Aus Brisbane | Biscuits | ? pesticide (wikipedia…) | Joy Ellen Thomas and another?; to change criminal court outcome | Criminal justice system & Arnott’s company | ? | Threat only? | Wasn’t convicted; real poisoned biscuits were posted and a massive recall prompted across 3000 supermarkets and service station outlets. Reported costs to Arnotts = $22 million. |
|  |  |  |  |  |  |  |  |  |
| Oct-Nov 1996 ^144,158^ | Dallas, TX | Pastries | Sigella dysenteriae T2 | Disgruntled employee = Diane Thompson | Coworkers | ?laced | 12 ill, 9 attended hospital, 4 hospitalised | TX Dept. of Health involved, “Epidemiologists interviewed 45 laboratory employees”; they found tampering with stored samples (missing 6 beads), lots of lab work to confirm same strain |
|  |  |  |  |  |  |  |  |  |
| Aug 1996 ^144^ | Japan | Bakery products | E. Coli | Tokiyuki Asaoka, extortion | Yamazaki Bakery | Injected | Threat only (letter) | Confessed to extortion; additional info may be minimal |
|  |  |  |  |  |  |  |  |  |
| May 1996 ^144,159^ | Britain | Milk products | Micro-organisms | Michael Just, microbiologist with debts; extortion | 5 big companies | Threatening letters | Threats only | They paid up; he was caught when he tried to cash in; he had real Yersinia enterocolinica which can cause digestion problems; Carus says it’s unusual for extortionists to have real bugs |
|  |  |  |  |  |  |  |  |  |
| Jan+ 1996 ^144^ | Essen, Germany | ? | Snake venom | Extortionists; wanted 400m dmarks | Food retailers? | ? | Threat only | Meetings were held with 60 European food manufacturers to discuss the threat; no further info |
|  |  |  |  |  |  |  |  |  |
| 1995^49^ | USA | Rice-a-roni | Insecticide | Donald William Bradley; extortion | Supermarket | Laced | Threat & plot only? | ? |
|  |  |  |  |  |  |  |  |  |
| 1995^160,161^ | China | Cooking ingredts, veg, meat, porridge | Rat poison | Du runqiong & son=Tang Youhua; superstitian | Neighbours | Spread, snuck into homes or on market foods | 18 died, 163 more ill that went to hospital | Had poisoned rice & tea in her house, too, rampaged for months, killing animals as well. Pair executed in Jan 1996. Poisoned wildlife & domestic animals, poisoned local pond. |
|  |  |  |  |  |  |  |  |  |
| Nov 1995 ^144,162^ | UK | Food | HIV infected blood | Barry Dixon (employee?); extortion | Sainsbury’s | ? | Threat only | Received 4 yr sentence for extortion. Not clear if he made it clear the blood would get into food. |
|  |  |  |  |  |  |  |  |  |
| Oct 1995 ^163^ | Dudley, WMids | Food | Aids | Frank Riolfo, extortion | Phone call & ? to Tesco | ?; black ink was injected into items | Threat only | He was jailed for 8 yrs; his wife acquitted of abetting |
|  |  |  |  |  |  |  |  |  |
| 1995^95,104^ | Tajikistan | Champagne | Cyanide | ?; political motives suspected | Russian soldiers | ? | About 10 died | ? |
|  |  |  |  |  |  |  |  |  |
| ? Feb 1995^49^ | Singapore | Sugar | Cyanide | ?; not identified | ? | ? | Implied some taken ill | Only article is unavailable |
|  |  |  |  |  |  |  |  |  |
| Feb of ? 1995^164^ | Edinburgh Scotland | Fruit juice (2 bottles); huge dose required to kill | Weedkiller | Wayne Smith; schizophrenic, God told him; some form of extortion | Police | Poisoned | ? threat & plot only; 1 bottle sold & returned without consumption | In 2 bottles then phoned shop to put in his threat, sent letters to police that police soon proclaimed a hoax. Also initially confessed to Paul Agutter’s crime (below). & they shared prison cell, too. |
|  |  |  |  |  |  |  |  |  |
| Dec 1994^165^ | Vancouver British Colombia | Frozen turkeys | Rat poison | Animal Rights Militia; political | ? | Injected | Threat only, no poison found in samples, no one ate | Thousands of turkeys pulled from shelves in metropolitan area after anon. letter to 2 supermarket chains; dead chicken sent to TV station. Big economic cost; turkeys quickly restocked. |
|  |  |  |  |  |  |  |  |  |
| Jan 1994^144^ | UK | Food | HIV, bacteria, chemicals | Michael Norman, Alexander Taylor, extortion | Tesco, Sainsburys, Safeway | ? infected fluids | Threat only | Sent letters; jailed for 5 & 8 yrs. |
|  |  |  |  |  |  |  |  |  |
| ? 1993-1994^49^ | Hong Kong | Vita Lemon or chrysanthemum tea | Rohypnol (sleeping drug) | Kwok Yuk-ho; robbery | via convenience stores | Injected | ? mostly mild | He would follow them out of the stores & take their wallets when they were incapacitated. |
|  |  |  |  |  |  |  |  |  |
| 1993^49^ | Cyprus | ‘food’ | Acetone | Maria Martin; business rival | Rival business | Sprinkled | Plot only, caught in act | ? |
|  |  |  |  |  |  |  |  |  |
| 1993 ^166^ | Cameroon | Peanut sauce | Rat poison | Pierre Wazan; family rancour | His uncle | put | 15 dead + 12 hospitalised + more ill | Sauce was used as basis for products sold at outdoor market; uncle didn’t ingest any. |
|  |  |  |  |  |  |  |  |  |
| Late 1992 ^49^ | Edinburgh UK | Toast | Rat poison | 2 residents (boys age 14 & 15); ? | ? | Put | Plot only, noticed before eaten | ?was indiscriminate in execution |
|  |  |  |  |  |  |  |  |  |
| Nov-Dec 1992^167,168^ | Upstate NY, USA | Chocolate marzipan and another candy | Thallium (in rat poison product) | Filip Semey, Belgian student: lovers dispute? | Marie-Aline Stacanov | Laced | 4 ill, seems all recovered; another received poisoned items but didn’t eat | Charged with 5 counts of attempted murder, tried in Belgium to where he fled; would need French or Flemish language search to get more info |
|  |  |  |  |  |  |  |  |  |
| Sep 1992^169^ | Jerusalem, Israel | Ground meat, fresh produce, chicken soup mix | Rat poison, bug spray | Rashid Zahada; Arab working in E. J., political reasons? | Israeli shoppers | Sprinkled and injected | ? | Also spiked coffee of his boss with poison. Confessed to all these actions. |
|  |  |  |  |  |  |  |  |  |
| Sep 1992 ^144^ | UK | Food | HIV | ? aka “The Terminator” extortion | Budgens | ? | Threat only | Claimed to contaminate 9 products in 100 stores; never caught |
|  |  |  |  |  |  |  |  |  |
| Jun 1992^170,171^ | Zhengzhou, Central China | Wheat flour | Arsenic: 2-300+ lbs | Li Yuan; expelled student, complicated personal revenge | ? | Entered cafeteria by broken window & mixed 2+ lbs into flour | 788 ill, no deaths | Many required hospital treatment. 8 hospitals involved. Li Yuan confessed. |
|  |  |  |  |  |  |  |  |  |
| Jan 1992 ^144^ | Canada | Cold buster bars (snack) | AIDS, or possibly oven cleaner | Animal rights militia (different names)  Animal research | Manuf. | ? | Threat only | Cost $250,000 to company; not caught |
|  |  |  |  |  |  |  |  |  |
| Jan 1992^172^ | Thailand | Noodles | Pesticide | Sanong Jankoong; jealous of step-daughter | Daughter | Laced | D’ter + cousin died, other relatives ill but recovered | ? |
|  |  |  |  |  |  |  |  |  |
| August 1991 ^173^ | Charles Town, WV, USA | Vending machine soda | Strychnine (rat poison) | ? | ? | ? | Went to hospital but full recovery | Other cans in the vending machine checked & had no signs of tampering. “FDA investigators are inspecting stores and bottling plants in the area” No public recall, no naming of brand of soda or where purchased. No followup news. Other explanations quite plausible or targeted? |
|  |  |  |  |  |  |  |  |  |
| July 1991 ^101,102^ | Heibei, China | Rice | tetramethylenedisulfotetramine | ? | ? | Laced? | 78 affected badly, no deaths | Site investigation to find cause of poisoning. Blood tests to find the toxin in victims.  Cause identified within 44 hours. |
|  |  |  |  |  |  |  |  |  |
| 1991^174-176^ | NY State USA | Chocolate and sweets, gifts he gave to colleagues | Mercuric chloride (insecticide) | Gary Kosowsky; motive? | 5 colleagues | Injected | 3 ate & were ill, full recovery (?) | On probation for theft at time of attacks. Jailed for crime of attempted murder. After his release, mother Evelyn died in 2009 & wife Krista went missing and her body found in 2013, her death unknown cause (?). |
|  |  |  |  |  |  |  |  |  |
| 1991^49^ | UK | Ground beef | Rat poison (low dose) | James Rodwell; extortion | supermarket | Sprinkled | Threat only | called manager to make threat; dose too low to hurt people |
|  |  |  |  |  |  |  |  |  |
| 1991^49^ | Japan | Yakult | Pesticide | ?; extortion | Manufacturer | ? | Threat only, 2 packs poisoned | ? |
|  |  |  |  |  |  |  |  |  |
| 1991^49^ | Japan | Chocolates and sweets, bottled veg juice | Agric. Chemicals, insecticides | “Heavenly Heart”; extortion | Supermarkets | Laced, poisoned | Threat only, partly implemented | ?No other reports found. |
|  |  |  |  |  |  |  |  |  |
| 1991^49^ | Israel | Gerber baby food | Pesticide = Methomyl | ? | ? | ? | 1 baby ill | ? |
|  |  |  |  |  |  |  |  |  |
| April 1990^177^ | Oak Ridge TN | Popcorn, salt & peanuts | (potassium) cyanide | ? | ? | Laced; delivered to homes | 5 people received food items | No reports ingested; Feds warned all 5200 employees on site about poisoning danger. |
|  |  |  |  |  |  |  |  |  |
| 1990 ^138^ | Philippines | Pineapple | ? | Huk terrorists | ? | ? | Plot only | ? |
|  |  |  |  |  |  |  |  |  |
| 1990^49^ | Amsterdam NE | Night club drinks | ? | ? | ? | Spiked | 6 ill, one of whom died | ? |
|  |  |  |  |  |  |  |  |  |
| 1990^49^ | Jerusalem Israel | Milk | Paralhion | Fuad Assila; unhappy employee | ? colleague | Put | 1 person ill | ? |
|  |  |  |  |  |  |  |  |  |
| Late 1989 ^49^ | Jerusalem Israel | Cabbage, falafel | Parathion | Ziad Tumar; ? nationalism | ? | ? | 2 ill -> hospital, one seriously | ? |
|  |  |  |  |  |  |  |  |  |
| 1989 ^49^ | Chile/USA THREAT | Grapes | Cyanide | Opponents to Pinochet | Anybody (Chilean regime) | Injected | Threat only | Thousands of boxes of fruit were opened & checked; 2 grapes were poisoned, no further PH response? |
|  |  |  |  |  |  |  |  |  |
| April 1989, esp. 7-4-1989 ^178-184^ | UK and possibly Eire | Baby food jars (also pet foods in 1988) | Caustic soda (also glass shards, pins, razor blades) | Ex-policeman: Rodney Witchelo (and copycats); extortion, RW demanded £3.75 miln | Heinz, Cow&Gate | spiked | Reports vary, but at least 5 babies injured or monitored, and at least 2 adults cut by glass; no reports or harm from caustic soda (RW’s threat) | 2 jars from blackmailer (spiked with CS). Other many dozen other incidents were copycats, PH response was a lot about managing them, too. 1500 stores removed items from shelves. Heinz already had crisis management & comms strategy to calm panic, but police didn’t want publicity, while public angry they weren’t informed. Eventually Heinz destroyed all stock & replaced with tamper-proof containers. Public advised to hand inspect before feeding to babies (2 adults minor injuries this way). RW arrested, convicted 15-12-1990, 17 yr sentence, released 1998. Article on him inspired Robert Dyer (threatened to bomb supermarkets in ~2000). May have led to bar code scanning being introduced faster, too. |
|  |  |  |  |  |  |  |  |  |
| May 1989^144^ | Scotland | Foods | HIV-infected blood | Robin Smith, extortion | Supermarkets | ? | Threat only | “Police traced a telephone call that he made to one of the companies, and arrested him” |
|  |  |  |  |  |  |  |  |  |
| 1966-1989 ^49,185,186^ | USA | ? understood to be food or water | arsenic | Blanche Taylor Moore | Parents, husband, boyfriend | ? | 4 dead, one recovered; other victims suspected | Only caught when final husband was ailing. |
|  |  |  |  |  |  |  |  |  |
| ? Dec 1988 ^187^ | Sri Lanka | ? | ? | Woman; pressured by family for money | Her 7 children (? More) | Fed | 7 died | She committed suicide afterwards |
|  |  |  |  |  |  |  |  |  |
| Oct 1988 ^188,189^ | Florida | Soft drink | Thallium (rat poison) | George James Trepal, neighbour dispute | Whole family? | ? | 1 death + 6 other ill | Peggy Carr took 4 months to die. Police & epid. Investigation, arrest in 1989, convicted 1991, previous investigations for poisoning incidents, Trepal still on death row in 2011. Trepal was a chemist who also made crystal-meth. |
|  |  |  |  |  |  |  |  |  |
| July 1988 | Los Angeles | Orange juice cartons and plastic bottles, supermarkets | ? none; one test = cyanide, others neg. | ? ; possible copycats, no motive | ? | Punctured cartons, placed outside the fridges | None | No poison found, but notes next to the cartons to say they were poisoned. Products pulled from shelves, police, FDA & state health depts. involved, workers alerted to watch for tampering; consumers asked to return products |
|  |  |  |  |  |  |  |  |  |
| May 1988 ^190-193^ | Mostly Illinois USA, plus items she posted elsewhere in USA | Marshmallow or Rice crispy snacks plus milk(?) and juice (?cartons): drinks leaking & tasted bad, arsenic quite diluted, hence few ill | Arsenic, (? She also stole lead but most sources don’t’ say lead was in her ‘gifts’) | Laurie Dann ; spite (mentally ill) | ? | Personally delivered to most people she new (see right) & Sent 24 items thru post to others she knew | 6+ people treated & released for poisoning, at least 3 others ingested items with possibly lesser affects | Lots other property destruction, theft and mentally unstable behaviour, ending in shootings & hostage takings…. Already pursued by FBI by time she attacked with poisons. She made rice crispy snacks and prepared packaged juice drinks, apparently injecting them with arsenic, a syringe was later found in her room with traces of the poison.  She awoke early on May 20th, delivering her venomous treats to a couple of frat houses and six homes in Glencoe, Highland Park, and Winnetka. Arriving at her employer’s house to pick up the boys, she gave them tainted milk. The boys thought it tasted funny and threw it out while Laurie wasn't looking. |
|  |  |  |  |  |  |  |  |  |
| Sep 1987-May 1988 ^194^ | Japan | Chocolateor sweets | Various: ? acid; P cyanide; ? | ? | ? children | Left lying around | No stories of ingestion | At least 4 incidents, May have been copycat events |
|  |  |  |  |  |  |  |  |  |
| April 1988 ^195,196^ | Italy | Grapefruit | “Lethal poison” | “Organization of Metropolitan Proletariat and Oppressed Peoples”: political motives?? | ? | Injected | Threat only; tests showed harmless | Warnings to newspaper, police & govt offices, blue fluid found in grapefruits but final verdict = not poison. Public notified & warned including against juice, imports from Israel banned, grapefruits seized all over country after hundreds of tests. Group never heard from again (?) |
|  |  |  |  |  |  |  |  |  |
| 1988 ^49^ | Australia | Beer | Strychnine | Maxine Byram; motive? | ? | Laced | ? | No further info found; headline of cited article implies fairly serious impacts (life sentence). |
|  |  |  |  |  |  |  |  |  |
| 1983?-1987 ^197,198^ | USA | dessert, orange juice, pie, others | Arsenic or cyanide or both,; also hepatitis serum | Donald Harvey, serial killer: some ‘mercy killings’ | Patients, neighbour, lover’s family, x-lover | Laced | Unclear, but very many, including deaths | Severely abused as a child, DH Worked in hospital. Confessed to many poisoning events, hard to prove all. Committed many other attacks, including smothering & injecting poison to pts. |
|  |  |  |  |  |  |  |  |  |
| June-Aug 1987^199^ | Edinburgh | Coleslaw, grapefruit juice, Coca Cola, yogurt | Arsenic, ground glass, weedkiller=paraquat | Codename=Raven?; extortion | Safeway supermarket | Contaminated | ? threat only | 3 letters written demanding £40-£100k, wanted replies via personal ads in The Scotsman. Police investigation, 2 anon. phone calls with info, said “David Young” was culprit. Uniformed security guards deployed and extra examinations of delivered items by supermarket. |
|  |  |  |  |  |  |  |  |  |
| Feb 1987 ^200,201^ | NY Uni | Boxed valentines chocolates | atropine and pilocarpine and says one source Sparteine, all strong prescription medicines | John Buettner-Janusch; spite about drug sentence from judge; unclear why colleague or other contacts | Sentenceing judge & a prof. contact , plus 2 other unnamed contacts |  | Wives of the named men + a daughter were ill (one of the wives seriously ill) | He had been sentenced in 1980 by the judge for manufacturing illegal drugs; also was a well respected anthropologist.  Pled guilty to intention to harm. |
|  |  |  |  |  |  |  |  |  |
| 1976? - 1987 ^202,203^ | Kiev, Ukraine | ?; but had to be administered orally | Valium & solutions, Clerici (thallium based) | Tamara (& Nina) Ivanyutina (Ivana Yutina, Ivaniutina) (sisters); money or spite | Husbands, cousin, housemate, colleagues, children at school where TI worked |  | 40 poisoning incidents by family, 13 events resulted in deaths: 9 by TI. | TI caught in 1987 when she poisoned 9 people at school (her employer), including children who died. TI confessed to police but pleaded not guilty at trial due to her lack of education. Parents & Nina were also poisoners 1976-1987 (imprisoned) TI was last woman to be executed in USSR (Ukraine). |
|  |  |  |  |  |  |  |  |  |
| Jun, July 1987 ^49,204^ | Glasgow, Edinburgh, Reading UK | Grapefruit juice, yogurt, beverages | Arsenic, paraquat, ground glass | ?; extortion demands | Asda, Safeway | Spiked | A person injured from glass; otherwise threat only | Arsenic & paraquat were found in stated products; multiple incidents not clear if linked. |
|  |  |  |  |  |  |  |  |  |
| 10 Feb 1987^205^ | NY state, USA | Tea bag or cheese | Cyanide (only in the tea, not the cheese) | Dragljob S. Cetkovic, ? bizarre suicide plot?; to confirm lethal dose? | Supermarket | Laced | Threat only | Phoned supermarket to tell them about it; confessed, was under stress, got 5 yr sentence |
|  |  |  |  |  |  |  |  |  |
| Sep 1986 ^206-208^ | NJ, USA | Packet soup (Lipton cup-a-soup) | Cyanide (very high levels) | ? | ?not clear, couldn‘t rule out target = Louis J. Denber | Tampered | LJD died, no others detected; isolated incident | Just 2 of 5 packets in the box were contaminated. Only had a few sips saying it tasted wrong. Lipton pulled all such products from stores in the area, offered refunds for any return items, batch tracing, and stated they would consider ways to make it harder to tamper with the product on shelf; FDA tested 2300 packets at the same supermarket & more at other stores; FBI & police involved |
|  |  |  |  |  |  |  |  |  |
| 1986 ^138^ | South Africa | Fruit | ‘toxic chemical’ | Anti-apartheid, Azanian People’s Liberation Front | ? SA regime | ? | None discovered, threat only | Threat was published in Canada; 2 Canadian stores pulled SAn fruit from shelves |
|  |  |  |  |  |  |  |  |  |
| May 1986 ^49,209^ | Japan, Tokyo | Chocolate bars, yogurt, coffee | Cyanide, Insecticide | Unknown & Shigeru Yamashita, Tsuneo Kusaka and another; extortion | Supermarkets | Injected or laced | Found without anyone eating, had notes on them to say poisoned. | ? responsible for cyanide event. SY & TK caught after 2^nd^ event, when. Money was deposited after extortion letter received. 3 mentioned in arrest, little info more. Thought to be part of string of copycat events. |
|  |  |  |  |  |  |  |  |  |
| 1986^184^ | USA | Baby food jars | ? | ? | Gerber | ? | ? | Mentioned in report on Rodney Witchelo’s incident |
|  |  |  |  |  |  |  |  |  |
| 1985-1986 ^49,210,211^ | Australia | Baby food jars | Rat poison, caustic soda | Edward John Dowling; extortion | ? | ? | No one ingested | Mass product recall, got 20 yr sentence |
|  |  |  |  |  |  |  |  |  |
| Dec 1984^212^ | Grimsby & elsewhere UK | Frozen turkey | Weed killer | Animal rights group; ALF; political extortion | Supermarkets | Spiked | No one ingested, one turkey with mercury found (Grimsby) | Poison threats received at stores in London, Northampton & Coventry. Claimed poisoned turkeys marked with black X (only one found?) |
|  |  |  |  |  |  |  |  |  |
| Late 1984-1985^213^ | Tokyo, Japan | Beer (Soya sauce & vinegar @ restaurant); 3^rd^ case where no one charged, too. | Poison (sodium cyanide) | Terukuni Nakano, Michiko Hiroshima (and 4 others); extortion | Brewery (restaurant) | Threat only ;(spiked) | Threat only (waitress spotted tampered bottles) | Separate incidents (by target), considered Copycats of man-with-21-faces extortion |
|  |  |  |  |  |  |  |  |  |
| Sept 1984 ^214^ | Germany | Boxed chocolates | Strychnine | Milan Nekuda; for wife’s inheritance | Wife’s aunt | Laced | 1 friend of aunt’s friend was very ill | ? |
|  |  |  |  |  |  |  |  |  |
| June 1984 and later ^144,215,216^ | Quincy IL and many others | Donuts, crispy chicken, drinks, Tea | Arsenic, ant-poison | Michael Swango; motives unclear | colleagues | ? | 6+ were ill and many more suspected, linked deaths | Arrested and imprisoned; linked to many suspicious activities, especially poisonings of colleagues, active as late as 1993 |
|  |  |  |  |  |  |  |  |  |
| 1984 ^2,212^ | UK | Mars bars | “rat poison” | Animal rights group = ALF; political extortion | anyone | injected | Threat only, threat notes found in candy bars | “Animal Liberation Front : ALF”, later admitted to be a hoax. Halted Mars bar production at high cost to manuf & much police action, many pulled from shelves. Scotland Yd did analysis |
|  |  |  |  |  |  |  |  |  |
| 1984-1985 ^49,217^ | Osaka, Kyoto and Nagoya, Japan | MorinagaSweets, cookies | cyanide | Never caught, Extortion | 30+ companies | laced | No one consumed | Candies pulled from shelves & criminal investigation. Huge economic disruption for companies involved |
|  |  |  |  |  |  |  |  |  |
| 1984 ^217,218^ | Japan | Glico candy | Cyanide | Extortion | Anyone | ?laced | No one consumed, only threat | Candies were pulled from shelves. No evidence of actual poisoning |
|  |  |  |  |  |  |  |  |  |
| Aug & Oct 1984 ^144,219-221^ | The Dalles, Oregon | Salad | Salmonella Typhimurium (they researched many others) | Cult members = Rajneeshees; political | townspeople | Sprinkled on salad in 8-10 restaurants | 751 people ill in 2 outbreaks (no deaths) | Plenty PH response, Will be well detailed on proper pub, CDC very involved. Had other plans, but only discovered later, no PH response at time; Carus says this is only USA bioterrorism incident (to date) that ever resulted in human illness. |
|  |  |  |  |  |  |  |  |  |
| May 1984 ^222^ | Slidell LA USA | Stuffing mix (shake n bake) | Pesticide | ? | ? | ? | 1 box found without consumption | Warning letter sent which caused search for tampered product, but no details on motive; product removed from shelves of affected store & one other store, no others found. |
|  |  |  |  |  |  |  |  |  |
| May 1981 ^49^ | Northern England | Jarred food | ? | ?, extortion | 3 shops | Contaminated | ? no one ingested | Threatening letter sent & contaminated jars found |
|  |  |  |  |  |  |  |  |  |
| 1978-1980 ^144,223,224^ | Rhodesia | Live cattle | Anthrax | CIO= unit of Rhodesian govt. | Rebels | Cattle deliberately given anthrax disease | 10,783 ill, 182 died; probably inhaled not eaten, though | Maybe none by formal sources, only at ground level as people realised cattle had something they were passing to humans? may be patchy documented |
|  |  |  |  |  |  |  |  |  |
| 1978-1980 ^223,224^ | Rhodesia | Canned meat | Thallium | CIO= unit of Rhodesian govt. | Rebels | Injected into cans surreptitiously given to insurgents | Guerillas also gave meat to hungry villagers, many of whom died | None? |
|  |  |  |  |  |  |  |  |  |
| 1980-1981 ^225-227^ | IL, USA | Pea soup, milk, cookies, Café food, shared meals | Arsenic | Charles Albanese, wanted inheritances | 5 relatives | ?, detail may be buried in court reports, had close access | 3 died, 2 mostly recovered | Caught after investigation of brother who ended up in wheelchair and community fears about sudden deaths of others. Convicted in 1981, Executed in 1995. |
|  |  |  |  |  |  |  |  |  |
| Dec 1980 ^228-230^ | S. Lake Tahoe CA | Kool-Aid | Cyanide | ? | ? | ? | 3 children under 5,may have fully recovered | Very possible the 3 were targeted; tests did not find cyanide in other packets in box or in sugar mixed with the Kool-Aid. Rushed to hospital, UC Davis or Berkeley found the cyanide police investigated Cal. Dept of Health involved. Supermarket cleared all product but batch was found to be free of poison, very likely to some extent targeted or not deliberate … |
|  |  |  |  |  |  |  |  |  |
| 22 & 29 Mar 1980 ^231,232^ | Beaverton OR & San Diego CA USA | Jarred pickles, teriyaki sauce, salad dressing | Cyanide (SD) | Paul Baileaux, Oregon jeweller, extortion | Safeway stores CA & Fred Meyer store OR | Put. | No one ingested, real jars found | Threat phoned in, also Notes found taped to 2 jars (one each store), had said 5 jars. High doses cyanide found in contaminated jars. County Dept of health & FBI involved. Got 2 x concurrent 20 yr sentences. Fingerprints did not match RQW, probably via those that PB was convicted. No separate data re Fred Meyer extortion event. |
|  |  |  |  |  |  |  |  |  |
| 1978 or 1979 ^233^ | Sun City AZ USA | Supermarket jarred food? | ? | Richard Quincy Williams tried but acquitted, extortion msgs | ? | ? | ? | Acquitted May 1979 in Arizona plot, arrested and released in San Diego plot April 1980. Bailleaux convicted for SD incident. |
|  |  |  |  |  |  |  |  |  |
| Sept 1978 ^234,235^ | Omaha NE USA | Lemonade & milk | Dimethyl nitrosamine | Steven Roy Harper, jealous of ex-lover’s husband’s family | Neighbours | ? | 2 deaths, 3 ill | ‘Intensive’ police investigation. Employer that gave him access to the DNsamine was unsucc’ly sued for negligence and compensation. |
|  |  |  |  |  |  |  |  |  |
| 1974, 1978-79 ^236^, 1988 ^138,237^ | Israel | Citrus fruit exports | Liquid Mercury | ‘Arab Revolutionary Council’ | Israeli ec onomy | Injected | 12 people were “injured”, significant trade disruption | Fruit exports and sales were delayed or stopped while fruit was searched for mercury. More economic & criminal. 5 Dutch children were hospitalised (stomachs pumped) but recovered. 14 poisoned organs found in batch of 220, non-lethal doses. One Dutch supermarket announced plans to test all oranges before display for sale. |
|  |  |  |  |  |  |  |  |  |
| 1977-1984 ^238^ | Japan, multiple incidents | Vending and supermarket foods | Cyanide, agricultural chemicals, others? | ? | ? | Laced, etc. | Alludes to hundreds of incidents, Many ill, 13+ died | “In 1977 two high school students died after drinking cyanide-laced cola that had been placed in a Tokyo telephone booth, and immediately afterward tainted confectionery was found at Tokyo Station. In 1984 cyanide-laced snack foods were placed in supermarkets in the Kansai, Tokai, and Kanto regions, with several hundred copycat crimes being recorded altogether. And in 1984-85 there was a spate of crimes across the country in which drinks laced with agricultural chemicals were put in vending machines; 13 people died in this chain of incidents.” |
|  |  |  |  |  |  |  |  |  |
| 18 Nov 1978 ^239^ | Jonestown Guyana | Flavour-aid | Cyanide | Jim Jones & cult members, religious extremism | Followers | Mixed | Probably 100s | Evidence that many (ie, children) didn’t realise it was poisoned & others were held down and forced to drink it; could argue little PH response? |
|  |  |  |  |  |  |  |  |  |
| 1969-1977 ^240,241^ | USA, mostly NC | tea in one event, others unclear | Arsenic, strychnine (ant poison) possibly others | Velma Marie (Bullard) Barfield, drug addiction money | Family & employers | Placed | Min. 6 murders, possibly 3 others attacked but recovered | Not very much detail on investigation, daughter of one of her victims pressed for detailed autopsy which revealed the arsenic poisoning, some more detail in appeal judgements. |
| . Da |  |  |  |  |  |  |  |  |
| 13 Oct 1975 – 1 Jan 1977 ^144,242^ | Denver CO, TX USA | ?food products on shop shelves | ? | Stephen Grant Morton | Grocery stores; extortion | Placed | ? | He was indicted by grand jury but govt. requested that case be dismissed |
|  |  |  |  |  |  |  |  |  |
| Summer 1977 ^243^ | Creances France | Wine | Atropine (prescript drug) | Roland Roussel, revenge | Woman he thought responsible for mother’s death | Put | Uncle died and 3 others seriously ill | ? |
|  |  |  |  |  |  |  |  |  |
| 31 Oct 1974^244,245^ | Houston TX | Candy | Cyanide | Ronald Clark O’Bryan; wanted son’s life insurance $ | Timothy Marc O’Bryan (his son) | Laced | TMO died; 4 other children supplied with poisoned candy didn’t eat it | Lots of details on criminal investigation, trying to track down source in *Statesman*. Arrested by 5 November, Convicted and put to death. T-or-Ting stopped locally for yrs, people threw out the candy their kids had. |
|  |  |  |  |  |  |  |  |  |
| 16 Oct 1973 ^246^ | ? USA | Orange juice | Scopolamine ?; pharma drug to prevent nausea | Woman | ? | Pretended it was a tasting survey, whoever she found in shop | 3 ill, no deaths | In a jewelry store. 3 people ; rest of info behind paywall |
|  |  |  |  |  |  |  |  |  |
| 16 Oct 1972 ^247^ | Germany | Coffee | Diphenylhydantoin, epilepsy med | ? | ? | ? | 12 US Army pilots ill | Recovered within 24 hrs |
|  |  |  |  |  |  |  |  |  |
| 1961-1971 ^248,249^ | UK | Coffee, tea, drinks and ? | Thallium, antimony, belladonna, sugar soap | Co-worker=Graham Young, motive? | ?colleagues, stepmother, other family, fellow prisoners, & school mates | ? | 3 died, 3+ others only ill | “It was only when Young himself suggested to a visiting health expert that the cause might be thallium that the illness was correctly diagnosed. Young, a former Broadmoor patient, was found guilty of murder and sentenced to life imprisonment. He committed suicide in 1990 in Parkhurst prison.  The case was a milestone in forensic detection. At the Metropolitan Police forensic laboratories, the ashes of Robert Egle, one of Young's cremated victims, were analysed by a technique known as atomic absorption spectrometry, which revealed a level of 5 parts per million of thallium - proof that Mr Egle had been poisoned with it.” |
|  |  |  |  |  |  |  |  |  |
| 1970 ^144,250^ | QuebecCanada | Shared meal | Parasites: ascariasis | Room-mate, Eric Kranz; he was acquitted; revenge | 7, incl. 3 College room-mates | Contaminated | 2 severely ill, 4 hospital’d, all recovered | Wikipedia; Kranz was acquitted of deliberate poisoning so maybe shouldn’t be in here, but most authors seem to think he was acquitted on technicality/in political deal & that he was guilty (ie, Carus) |
|  |  |  |  |  |  |  |  |  |
| 1969 ^49^ | Peru | Group meal | ? | M Soto, ?motive | ? | ? | 8 died including self | ? |
|  |  |  |  |  |  |  |  |  |
| 1964-66 ^144^ | Japan | Cakes, milk, clams, fruit | Dysentery, typhoid, many events | Dr. Mitsuru Suzuki; power trip | Friends & colleagues & family | Laced or injected | Many ill, no deaths | The PH response would be all the investigations at so many small outbreaks of typhoid, etc. I won’t find back-stage details on how that evolved. (Also injected typhoid straight, and put in medicines or barium solution). This case features official cover-up by his university, knew he was poisoning ppl. There are several 1930s Japanese poisonings also described in Carus SW. |
|  |  |  |  |  |  |  |  |  |
| March 1961 ^251^ | Kuzuo, Nabari, Japan | Wine | Pesticide | Masaru Okunishi; complicate love life | Wife & mistress | (laced) | 5 died, including targets | Unclear but likely, will be buried in Japanese somewhere |
|  |  |  |  |  |  |  |  |  |
| 1957-1965 ^144,252^ | Rural Brazil | Sugar | Arsenic | Govt. service; political | Indigenous popn | ? | Unclear | Was part of a multi-faceted genocide campaign; original report “lost” but resurfaced in 2013 |
|  |  |  |  |  |  |  |  |  |
| 1954 ^253,254^ | Germany | Milk, yoghurt, chocolate truffles | Parathion (E605) | Christa Lehman, 2 spite + 1 accident | Family, neighbour | Intended family & unintended neighbour | 3 died, only 2 intentional | Tests were especially developed to detect E605 |
|  |  |  |  |  |  |  |  |  |
| 1920s-1954 ^255^ | USA | ? | Rat poison, others? | Nancy Hazel (Nannie Doss), money & ? | Many | ? | 11 deaths | ? |
|  |  |  |  |  |  |  |  |  |
| 1947-1953 ^256^ | Australia | Tea, maybe others? | Thallium | Caroline Grills, motive ? | Family members | ? | Up to 4 murders & 3 other attempts | There was a public health scare in the city about thallium poisonings at the time, possible she was doing distraction poisonings of random people. |
|  |  |  |  |  |  |  |  |  |
| 1952 ^104,141,144^ | Kenya | Cattle | African milk bush toxin | Mau Mau rebels; political | British colonists | ?orally? | 33 cattle poisoned, 8 died; unclear human impacts | Probably all economic/war, vet institute investigated the event not a PH body |
|  |  |  |  |  |  |  |  |  |
| April 1946 ^144,257,258^ | Nuremberg | Loaves of bread | Arsenic | Nakam or Nokmim, Jewish avengers | SS soldiers in POW camp | Arsenic spread on the loaves in bakery that supplied the camp | Thousands ill, hundreds died | Massive operation to treat victims and find cause, but unclear specifics |

**WATER ATTACKS**

| **When** | **Where** | **Type of (drinking) water** | **Contaminant (dose)** | **Who did**  **& Why** | **Who Intended** | **Method** | **Impact (who affected & how)** | **Response, esp. public health** |
| --- | --- | --- | --- | --- | --- | --- | --- | --- |
| Aug 2014 ^259^ & May 2015 ^260^ | Liberia | Well | Ebola | ? | ? | Dropped | Attempt witnessed, no casualties | 2014 2 men arrested.  2015 Well was closed down; could it be unfounded rumours? |
|  |  |  |  |  |  |  |  |  |
| 2015 ^261^ | Trinidad | Tap water | “oily substance” | ? unclear if deliberate | ? | ? | Reports of mild illness | Unclear, massive operation to get other water in & tell ppl not to drink |
|  |  |  |  |  |  |  |  |  |
| March 2014 ^262^ | Bourgin plant, Merida, Venezuela | Tap water | Diesel fuel | ? | ? | ? | no harm reported | Discovered by routine procedures, ample evidence of deliberateness, one source said someone was arrested |
|  |  |  |  |  |  |  |  |  |
| March? 2014 ^263^ | China, Yunnan, Hebei? | Well | Rat poison, tetramethylene-disulfotetramine | He Feng who ran rival Xiyangyang nursery | Children @ Jiajia nursery | Somehow added | 76 children hospitalised, 2+ died | 'By the time the emergency services arrived a further six children had been taken ill and a full-scale emergency was announced.' |
|  |  |  |  |  |  |  |  |  |
| 2012-13 ^264,265^ | New York or near | ? or food | ? bacteria? | Ahmed Abassi, terrorist, with chiheb Esseghaier & Raed Jaser | ? | ? | Plot only, didn’t get past planning stages; Deported to Tunisia | Heavily investigated by FBI : “charged with two counts of knowingly making false statements to immigration authorities to carry out an act of international terrorism.” |
|  |  |  |  |  |  |  |  |  |
| Dec 2012 ^266^ | Lattakia, Syria | Water supplies | ? poison | Free SyrianArmy | Their enemies | ? | Threat only | Threat made In youtube video |
|  |  |  |  |  |  |  |  |  |
| Sep 2012 ^267^ | Kismayu, Somalia | Well | Poison | Al Shabaab; political | ? | dropped | No reports of casualties | Water and food supplies were airlifted in by Kenya Defence force; subsequent suspicious poisonings in 2014 same city |
|  |  |  |  |  |  |  |  |  |
| April 2012 ^18^ | TakharWarza HS, Khost Afgnstan | Drinking water | ? | ? not claimed | ? | ? | 250+ to hospital | To hospital, (Ismail Khil). Similar event on girls in 2012 said to be hysteria (?) |
|  |  |  |  |  |  |  |  |  |
| 2011 ^268^ | Jakarta Indonesia | Water and food supplies, police cafeteria | cyanide | 7 members radical group = Abu Ja’far, Ali Miftah, Anang and Furqon | Police | ? | Plot only, although hint of test killings | 7 men Convicted & sentenced to jail in April 2012: political motives |
|  |  |  |  |  |  |  |  |  |
| 2011 ^269^ | Libya | Civilian water plant | Al phosphide found at plant | Alleged Kadafi forces | Civilians | ? fear that was adulturated | 2 ppl to hospital, coincidence? | “Rebel leaders at some checkpoints close to Misurata have warned civilians not to drink tap water until tests have been conducted.” Unicef sent water trucks and bottled water |
|  |  |  |  |  |  |  |  |  |
| 2009 ^270^ | Taichung, Taiwan | Uni & school water supplies | ? rat poison | Wang Chiang-Sheng, extortion | ? | ? | Wrote threatening letters, threat & extortion for money only | Police investigation, there was a bag of rat poison placed by W C-S hanging in a water tank |
|  |  |  |  |  |  |  |  |  |
| 2004 & 2009 ^271^ | Thai-Myanmar border | Refugee camp water supply | Herbicides | ?; anti-refugee | Camp residents | ? | Plot only, (probably) discovered both times | Water brought from other sources, arrest in 2004, not caught in 2009 incident |
|  |  |  |  |  |  |  |  |  |
| Late 2009 ^272^ | Pakistan | Various water resources | 200 l of Poison | Tehrik-e Taliban | ? | Contaminate | Threatening letters only | ?; wanted Army to stop operations in S. Waziristan |
|  |  |  |  |  |  |  |  |  |
| 2009 ^273,274^ | Moscow | City | Poison | ‘terrorists’ | ? | ? | Plot only | Police were warned because “According to the intelligence agencies' information, the terrorists have prepared detailed maps of the city's water systems.” |
|  |  |  |  |  |  |  |  |  |
| December 2008 ^275^ | Mbabane, Swaziland | Water tank | Poison | Jealous man | Neighbours | Put | Discovered before consumed (plot only) | No one arrested; police investigated & water company tested… somewhat unconfirmed this |
|  |  |  |  |  |  |  |  |  |
| 2008 ^276^ | Varney WV | Drink water supply | 2 cannisters Cyanide | Roy Tiller | ? | Poison | ? plot only | Arrested, no further info or even why he was arrested. |
|  |  |  |  |  |  |  |  |  |
| By 2008 ^277^ | Europe, esp GB & Denmark | Water resources | Chemical agents (many listed) | Jihadis; retaliation for Prophet M. cartoons & other political motives | civilians | many proposed | Plot only (discussed on internet fora!), not clear how developed; debated and opposed by some | Unclear, may be closely related to 2007 UK reports below |
|  |  |  |  |  |  |  |  |  |
| 2007 ^278,279^ | UK | Reservoirs & treatment works | Poison | ‘terrorists’; Al Qaeda in one report | ? | Poison | (Threat or) plot only | Maybe multiple plots; Newspaper says Publicity blackout ordered to prevent panic; water companies ordered to increase security and alertness. “Security sources said that water companies received regular advice but there had not been any specific intelligence from last summer that terrorists were targeting the water supply.”  *More info behind paywalls* |
|  |  |  |  |  |  |  |  |  |
| 2004 – 2007 ^280,281^ | Toronto, Canada | Bottled water | Ricin | Adel Arnaout, for money | ? | Injected | H2O too foul to fool anyone | Charged with attempted murder, Ongoing investigation…; PH response very unclear |
|  |  |  |  |  |  |  |  |  |
| 6 Oct 2006 ^282^ | Greve, Denmark | Reservoir (drink water) | Strychnine = rat poison | Cover was smashed open | ? | Single vial of rat poison in water | ? (plot only) | Unlikely to be enough to cause harm; existing security measures were many. |
|  |  |  |  |  |  |  |  |  |
| 31 July 2006 ^283^ | Tring, England | Storage reservoir (a large tank) | Weed killer, Sodium chlorate | Break-in & contamination discovered by water co. staff | ? | ? | Anonymous tip off to local newspaper | Supply was shut off (and other?). Investigation by EA, DWI, Thames Water, Thames Valley Police. Tests confirmed the levels were not dangerous. |
|  |  |  |  |  |  |  |  |  |
| Jun 1999 ^274^, 2006 ^284,285^ | UK | Tap water | Paraquat (1999); unclear other dates | SNLA, violent independence; ?Adam Busby? | Not Scots | Poisoning Glasgow fire hydrants; other supplies in England | No info | No info |
|  |  |  |  |  |  |  |  |  |
| Early 2004-Sep 2005+ ^77,79,286-288^ | Italy. Treviso, Padua, Milan, other towns | Bottled water, other?? | Bleach mostly, also caustic soda | ?. Likely copycats of 2003 “Acquabomber” | ? | Injected (visible), but where in supply chain unclear; more often cafes/machines than on shelves? | 5+ people taken to hospital on 5+ occasions | Minimum 5 attacks ingested by 5+ people in 5+ places, likely copycats of initial 2003 water bottle attacker(s). |
|  |  |  |  |  |  |  |  |  |
| 4 May 2005 ^289^ | Kaikoura NZ | Reservoir supplying town | 1080 pesticide | ? | ? | ? | Threat only: graffiti discovered next to pellets of 1080 & broken manhole cover | Reservoir was drained; water was sent for testing, health authorities & community & police were notified & were investigating |
|  |  |  |  |  |  |  |  |  |
| Before 2001-2004 ^274,290^ | USA | Water treatment facilities | Poison | (terrorists) | ? | ? | Plot only | Reported in leaked FBI document; plot was only at recruitment stage |
|  |  |  |  |  |  |  |  |  |
| 2003-2004 ^274,291^ | Sudan | Drinking water wells | ? poison | Sudanese govt | Civilians | Poisoning | unclear | Unclear situation, extent or reality |
|  |  |  |  |  |  |  |  |  |
| 2003 ^142^ | USA | Tap water, http://the_ricin_solution.tripod.com/ | Ricin | ‘fallen angel’; labour rights dispute | ? | ‘dump’ | Threat only | FBI investigated. Not caught |
|  |  |  |  |  |  |  |  |  |
| 2003 ++? ^288,292,293^ | Italy | Bottled water | Bleach/ ammonia, acetone | ? original “Acquabomber” (see 2004-05 entry) | Anyone | Injected in lid, sometimes obviously | 30 ppl hospitalised, 20+ cities affected; | Bottles pulled from shelves & security guards posted; publicity to tell ppl what to look for; may have sparked copycats in 2004-05+ (treating copycats as separate from original series) |
|  |  |  |  |  |  |  |  |  |
| 2003 ^291,294^ | Jordan | Water supply to US troops | ? | Iraqi agents | American troops | ? | Plot only | Jordanians arrested |
|  |  |  |  |  |  |  |  |  |
| 2003 ^295^ | Henan, China | Reservoir | 500 ml of pesticide | Cao Qian, financial | ? | Tossed in | 64 ill, 42 hospitalised | Odd small dose so powerful; he wanted to drive up sales of his water purifying devices |
|  |  |  |  |  |  |  |  |  |
| 2002 ^296^ | France | ? suitable for water supplies | ? unstated, but said to be suitable for water contamination | 4 terrorists suspects | ? | ? | Plot only; they had chemicals & protective suit | Arrested by security services |
|  |  |  |  |  |  |  |  |  |
| 2002^297^ | South Africa | Township water supplies | Tetranium, agric. Poison | Boere Vryheids Aksie (BVA, rt wing extremists) | Black South Africans | ? | Plot only | Arrested by security services |
|  |  |  |  |  |  |  |  |  |
| 2002 ^298^ | Denver CO | Any municipal | ? | James & Mustafa Ujaama, Semi Osman & Al Qaeda operatives | ? | ? | Plot only (or perhaps instruction book only); part of Abu Hamza saga | FBI arrested the men, Ujaama brothers convicted, one gave lots evidence. Were involved in many plots, but water connection probably tenuous (may have had info but no formulated plot) |
|  |  |  |  |  |  |  |  |  |
| 2002 ^291,299^ | Winter Park, CO USA | Tap water tanks | ? | Earth Lib. Front, env. Extremists | ? | ? | Threatening letters only | FBI involved |
|  |  |  |  |  |  |  |  |  |
| 2002 ^274,291,300^ | Rome, Italy | Tap water supply in a commercial area of city, including US embassey | Cyanide-based: potassium ferrocyanide | 4 + Moroccans | ? | ? | Threat only | 4-14 people arrested; suggested links to Al Quaida, ineffectual poison |
|  |  |  |  |  |  |  |  |  |
| 31 Dec 2001 ^49^ | Singapore | Bottle mineral water | Methomyl (pesticide) | Quek Loo Ming, unclear motive | Doreen Lum | Poured in | Fong Oi Lin died, 2 others ill | unavailable |
|  |  |  |  |  |  |  |  |  |
| 2001 ^280,291^ | Philippines | Supply | ? poison | Abu Sayyaf Islamic militant separatists | Christian town = Isabela, Basilan | Poison | Threat only | villages suspected contamination due to water that smelled like gasoline. Local officials responded by closing pipelines and bringing in drinking water by truck, may be rumour more than plot |
|  |  |  |  |  |  |  |  |  |
| 2000 ^291^ | France/ Belgium | Meuse River tributary; Meuse is drink water source in The Netherlands | 5000 litres sulphuric acid | Chemical plant workers | Anyone | Dumped It | Fireman stopped it reaching river = PH response | Firemen, AND “Local authorities evacuated the entire area within a 500-meter radius of the factory” |
|  |  |  |  |  |  |  |  |  |
| 1999 ^301^ | England | Water supply via fire hydrants | poison | ?man arrested by Irish police; wanted British troops out of NI | Public | into | Plot only | Publicity suppressed.  Britain's Cabinet office said the threat "displayed enough technical detail to give cause for concern." |
|  |  |  |  |  |  |  |  |  |
| 1999 ^291^ | Kosovo | Drinking water wells | Dead human bodies and other hazardous substances | Serbs | ? | Thrown in | ? | ? |
|  |  |  |  |  |  |  |  |  |
| 1999 ^49^ | Kagoshima, Japan | Hot water for office drinks | Arsenite | ? | ? | In | 5 people taken to hospital | Police thought deliberate |
|  |  |  |  |  |  |  |  |  |
| 1999 ^291^ | East Timor | Drinking water well | Dead human bodies | Militia opposing East Timor independence | ? | Thrown in | ? | ? |
|  |  |  |  |  |  |  |  |  |
| 1999^49^ | New Zealand | Town water supply | Superphosphate fertiliser | ? | ? | ? | Plot only; padlock to facility cut & fertiliser in | “Results showed contamination was minimal, no health risks to people.” |
|  |  |  |  |  |  |  |  |  |
| 1999 ^291^ | Central Angola | Drinking water wells | 100 dead bodies | ? | ? | ? | ? | ? |
|  |  |  |  |  |  |  |  |  |
| 1998 ^49^ | Exeter Uni, UK | Bio. Science tea kettle | mercury | William Smith (employee), ? | ? | put | Plot only(?) | ? |
|  |  |  |  |  |  |  |  |  |
| 1998^49,302^ | Hong Kong | ‘magic water’: Dalziel says K-sodium, but other sources say cyanide | Potassium cyanide (probably) | Li Yuhui, financial. Disputed if deliberate | ? | Mixed | 5 killed, no others ill? | Information online varies. Fortune teller giving ‘magic water’ with poison mixed in. Was sentenced to death. |
|  |  |  |  |  |  |  |  |  |
| 1998^35,303^  Aug  16 Oct  27 Oct  28 Oct | Niigata Japan  Mie University  Okazaki Nat. Res. Instititute  Utano Hospital, Kyoto | Teapot  Tea  Tea  Hot water | Sodium azide (all below) | ? | ? | Added | 10 drink, 9 ill  4ill  6 ill  8 ill. 4^th^ incident in 3 months | ?Niigata = timber processing company. Others were research institutes or universities or hospital |
|  |  |  |  |  |  |  |  |  |
| 28 June 1995 ^304,305^ | Cancer lab at US NIH Bethsheda MA | Office water cooler | Phosphorous-32 | ?, accepted as deliberate | ? Maryann Wenli Ma & 26 others | ? | Vomiting & pain, she was pregnant at time of ingestion. Otherwise Not clear, this may be in scientific lit. Some dispute on facts in this incident (!) | FBI investigated…Since the incident, NIH has "made significant efforts to improve its control of radioactive material," said NRC. "NIH has tightened its standards for the security and use of radioactive materials," noted NIH deputy director for intramural research Dr. Michael Gottesman. |
|  |  |  |  |  |  |  |  |  |
| August 1994^306-309^ | Edinburgh, Scotland | Tonic water, supermarket shelves | Atropine; caught because wife’s bottle had much higher dose than others | Paul Agutter, to cover up crime = biochem lecturer. Depression & Fin worries too | His wife; spiked her gin & tonic @ home; plan to marry Lover (they had open marriage) | Laced, poured | 8 ill including wife & own child; 4 of those hospitalised. One of two “The Safeway poisoner” s | Witnessed @ supermarket & “arrested after CCTV footage showed him placing the poisoned tonic on the shelf of an Edinburgh branch of Safeway.”… “sparked a nationwide alert”, 55,000 x 2 litre bottles withdrawn. 12 yr sentence. . 6-7 bottles found with poison |
|  |  |  |  |  |  |  |  |  |
| 1994 ^274,291^ | Tiraspol, Moldova | Munic. Tap water supply | Mercury | Moldavian General Nikolay Matveyev | Russian 14th Army | ? | Threat only | Some dispute, probably |
|  |  |  |  |  |  |  |  |  |
| Late 1993^310^ | Cotabato, Philippines | Deep well | ? | Retreating Muslim rebels | ? incoming troops | ? | 3 soldiers died, 25 more ill | ? Thirsty soldiers quickly succumbed, evacuated to nearby hospital. |
|  |  |  |  |  |  |  |  |  |
| 1992 ^274,291,311^ | Istanbul, Turkey | Military water Storage Tanks | Potassium cyanide | PKK | Turkish Air Force | ? | ? | No details (tried hard) |
|  |  |  |  |  |  |  |  |  |
| 1991 ^274,291^ | Kelowna, BC | Municipal tap water supply | Biological contaminants | ?; mentioned Gulf war in threat letter | ? | ? | Threat only | Security increased, no realised attack, threatener not found; |
|  |  |  |  |  |  |  |  |  |
| 1990s ^274^ | Washington DC | Tap water supply | VX, like sarin | ? | ? | ? | Threat only, Kroll says multiple threats | “These threats were taken so seriously that the govt. commissioned and funded into a dedicated online VX detector. Reports indicated that several detectors were built and installed in various secret key locations” (no reference any of that) |
|  |  |  |  |  |  |  |  |  |
| 1990^49^ | Israel | Roof tank water | Insecticide | ? nationalists?? | ? | Laced | 2 roof top tanks found to be tampered with; minimal info. | ? |
|  |  |  |  |  |  |  |  |  |
| 1990 ^144,312,313^ | Edinburgh, Scotland | Roof attic water tanks, urban area | Human faeces, incl. giardial cysts | ? | ? | Dumped some in | 9 ill in Giardia outbreak | Investigation as to cause; some dispute whether this was deliberate or vandalism without intent to cause harm to health |
|  |  |  |  |  |  |  |  |  |
| 29 Dec 1989 ^314^ | Sibiu (?Nadlac and other ?) towns in Romania | Town water supplies | Nerve gas (sarin, VX) | Romanian Govt. | Towns people | Dumped | Five ppl very ill, no reports of deaths | MSF doctors attended the ill; said that tests confirmed what was in the water. water drained & pipes refilled. Some dispute over whether this incident was real. |
|  |  |  |  |  |  |  |  |  |
| Aug 1989 ^144,315^ | Dobra, Namibia | Water supply at refugee camp = Dobra | Cholera, yellow fever | Petrus Jacobus Botes, acting on behalf of govt. | Camp residents | ? | Seems they did contaminate, but chlorine killed enough bugs to prevent impacts | None, story emerged subsequently; other materials and allegations during Truth & Rec. Commission, but not as clearly targeted |
|  |  |  |  |  |  |  |  |  |
| 21 Jun 1989^316^ | US FDA office, NYC | Office water cooler | Cyanide | Hector Cabassa; spite | His boss | Put | Someone else was ill instead (recovered) | Arrested, confessed, charged, subsequent outcome unclear |
|  |  |  |  |  |  |  |  |  |
| June 1989 ^142^ | FDA offices, NY | Office cooler | Cyanide | Cleaning worker | ? | In water) | One person ill | ? |
|  |  |  |  |  |  |  |  |  |
| Sept 1987 ^49,317^ | Sydney, Australia | Building water supply | Sheep dip (pesticide) | ? | ? | Put | 5 ill; no fatalities reported | Minimal info |
|  |  |  |  |  |  |  |  |  |
| 1987 ^104,274,318,319^ | Philippines | Drinking water and sweets | pesticide | ? | Police | ?ice water in plastic bags during fun run | 19 recruits died, 140+ hospitalised | ? not irrefutably proven deliberate |
|  |  |  |  |  |  |  |  |  |
| 1983-87, June 1990 ^104,144^ | Sri Lanka | Water supplies of the army | ? + chlorine | Tamil militants | Sri Lanka army | ? | ? Threat only 1983-87; 1990 1 died & 60 ill | Claimed to have an ongoing programme, patchy evidence |
|  |  |  |  |  |  |  |  |  |
| 1986 ^274,320^ | Large USA cities | Municipal water | Potassium cyanide (200 lbs pure) | James Ellison, of the CSA ; (Christian extremists) | Not white Christians | ? | Raided before used, Plot only | FBI recovered lots of Potassium cyanide from heavily armed camp (big siege says Wikipedia). Ellison testified against others, big trial in 1988. |
|  |  |  |  |  |  |  |  |  |
| 1985 ^274,319,321^ | NY City | City tap water | Plutonium | ?; wanted charges dropped against Bernard Goetz | ? | ? | Threat only | Plutonium levels were found to be 200x higher than expected, but still 0.4% of level high enough to be a health threat |
|  |  |  |  |  |  |  |  |  |
| 1985 ^291,319^ | Arkansas / NYC, W DC, Chicago, | Tap water supplies | 30 drums of potassium cyanide | Survivalist group in AR; religious motives | Sinners | ? | Threat only | ? |
|  |  |  |  |  |  |  |  |  |
| 1984 ^291^ | Oregon, USA | Glasses of drinking water | Salmonella typhimurium | Followers of Rajneesh cult; political | Voting population | ? | 2 local govt officials | Criminal investigation |
|  |  |  |  |  |  |  |  |  |
| 1983 ^274^ | Louisiana | Tap water supply | Cyanide | ? | ? | ? | ? | Traces of cyanide found, other actions? |
|  |  |  |  |  |  |  |  |  |
| 1983 ^291^ | Israel | Water in Galilee | Unidentified powder | Israeli Arabs | ? | ? | Threat only | Israeli govt. announced plot uncovered |
|  |  |  |  |  |  |  |  |  |
| 1982 ^274,291^ | Los Angeles | Tap water supply | Biological agent | Man | ? | Poison? | Threat only | FBI arrested |
|  |  |  |  |  |  |  |  |  |
| 1980 ^274^ | Lake Tahoe | Casino water supply | ? | ?, extortion | Casino | ? | Threat only | ? |
|  |  |  |  |  |  |  |  |  |
| 1980 ^319,322^ | Pittsburgh PA | Tap water. | Chlordane (pesticide) | ?; employment dispute? | ? | ‘injected’ | 150 ill | Concurrent with labour dispute, required specialist knowledge to inject in right place; Conf paper on difficulties of flushing water out |
|  |  |  |  |  |  |  |  |  |
| 1978-1980^223,224^ | Rhodesia | Watercourses | Cholera, | CIO= unit of Rhodesian govt. | Any rebel | Dumping cholera in water supplies, esp. the Ruya River | Cholera outbreak in Mozambique | none? minimal PH infrastructure, anyway |
|  |  |  |  |  |  |  |  |  |
| 1978-1980^224^ | Rhodesia | Wells and other water sources | Unknown poisons | CIO= unit of Rhodesian govt. | Any rebel | Directly added to source | Min. 200 deaths | Only local awareness? minimal PH infrastructure, anyway |
|  |  |  |  |  |  |  |  |  |
| 1978 ^144^ | Tuscon, Arizona | Drinking water system/fountains | typhoid | Never found out | City population | An anonymous letter was sent to the mayor demanding money and threatening the action | Threat only | FBI called in. Contingency plan was to withdraw all water from the wells and to procure stocks of chlorine (at the time the city supply was not chlorinated). |
|  |  |  |  |  |  |  |  |  |
| 1977 ^274,280,291^ | USA | N. Carolina reservoir | Unknown | ? | Anyone?? | Poured in?? | Disruption? | Contamination of a North Carolina reservoir with unknown materials.  According to Clark (who is CLARK?): “Safety caps and valves were removed, and poison  chemicals were sent into the reservoir....Water had to be brought in.” |
|  |  |  |  |  |  |  |  |  |
| 1975 ^144^ | Threat to Paris, Madrid, Rabat, Nouakchott | Water supplies | cholera | ETA (Spanish terrorist group) | General populations | Not specified | Threat Only | None |
|  |  |  |  |  |  |  |  |  |
| 1974 ^144^ | Jordan | Jordan River | Bacteria | ‘Middle East Firm’ | ? | ? | Plot only | ? (Poor quality evidence) |
|  |  |  |  |  |  |  |  |  |
| 1973 ^144,274,291^ | Germany | Tap Water supplies | bacilli of anthrax and  botulinum | Male biologist; extortion or anti-Jewish(?) | ? | ? | Threat only, wanted $8.5 million | “Police guards were placed around water reservoirs and regular water samples were taken. …Germans mounted a massive manhunt to track down the perpetrators. The German public reacted by buying large quantities of bottled water.” *Carus suggests this may have been a hoax* |
|  |  |  |  |  |  |  |  |  |
| 1972 ^274,291^ | NY City | Tap water supply | Nerve gas | ? | ? | ? | Threat only | Very little more info |
|  |  |  |  |  |  |  |  |  |
| 1972 ^144,291,323^ | US cities | City water supplies, esp. St. Louis & Chicago | Typhoid bacteria and other pathogens | “Order of the Rising Sun”? or RISE = diverse extremist goals | ? many/any | ? | Plot only | Police investigated ; two ppl were arrested and prosecuted = Allan Schwander & Stephen Pera |
|  |  |  |  |  |  |  |  |  |
| 1970, ^144,274,291^ | USA | Tap water supply systems | Biological agents | ‘The Weather Underground Org (WUO) or The Weathermen’; anti Vietnam/imperialism protest | ? | Contamination | Threat only | ? very little info available |
|  |  |  |  |  |  |  |  |  |
| 1948-2013 | Israel | Any drinking supply | various | Nationalists & Israeli govt (very alleged) | Non citizens | Various allegations, in news reports & books, not substantiated in scientific lit | Non-Israeli citizens | ? Disputed, no independent source |
|  |  |  |  |  |  |  |  |  |
| 1947 ^144^ | Germany | City drinking water | Poison | Nakam; Holocaust revenge | Everyone | ? | Plot only | Found out after successful bakery poisoning; Carus details quite a few accounts of other part developed plots |

**OTHER PEOPLE’S INCLUSION CRITERIA, and RELEVANT DATA**:

MOHTADI & MURSHID: Compiled 448 observations on CBRN events… said they “consulted” various databases & sources, but not clear that it was a systematic search or even what the full search strategy was, included acts of sabotage, direct attacks on facilities that contained relevant material, politically motivated or not: 25,594 incidents 1968-30 Jan 2006; excluded are hoaxes and pranks

GLEICK: goes into 2006; “Examples of violence related to both absolute and relative deprivation of water”: refers to book he published, and worldwater.org. Does not say it’s meant to be a thorough inventory

CARUS 2002: pg 37, names various sources and mentions local newspaper reports, but Not How they were searched for or found. Says his friends told him about some, that kind of thing; 1998 publish date with Feb 2001 revision, so roughly thru early 2000; all cases are described in this document and sources also used by M&M

DALZIEL 2009: 1950-2008, CBRN & non-CBRN, as long as malicious: includes any kind of poisoning of water or food: search strategy = first looked at Mohtadi/Carus/similar, then referred to specific reports & terrorism databases, no structured validity analysis. No open Internet search. Identifies points in production (HACCP style). Actual but not threats.

Gleick gives details of loads of water related attacks. Bomb events in Iraq 2003, Sudan, Pakistan, Kashmir and Sri Lanka not in table above.

Kroll ^274^ gives details of water threat events, too, especially 1970-2005.

List of events here: http://www.cancerletter.com/articles/20131204_11

More events detailed these places: https://www.jewishvirtuallibrary.org/jsource/Terrorism/chemterror.html

http://www.eupolis.regione.lombardia.it/shared/ccurl/894/59/ReportDefEN.pdf

“WSJ From 2000 to 2010, there were just 130 murders involving poison of all sorts.”: must be USA

***EXAMPLES OF INELIGIBLE EVENTS, USUALLY BECAUSE OF SMALL NUMBER OF ONLY TARGETED VICTIMS:***

| Nov 2006 ^324-328^ | London UK | Tea, pot was heavily contaminated | Polonium 210 (very high) | ? political | ? Alexander Litvinenko | Ingested (drank) | AL died soon, lots investigated | 33k passengers from 21 flights contacted. 5 aeroplanes id’d, 1 BA aircraft shown to be contaminated. 139 ppl referred to HPA, with 24 referred to specialist clinic. 24 venues monitored & contamination found at 12 of them. 53 healthcare staff plus 140 ppl who passed nr AL asked to provide urine samples. 2 hospitals involved. Quite a detailed account of investigation of others exposed. |
| --- | --- | --- | --- | --- | --- | --- | --- | --- |

This was proclaimed to be hysteria, although ongoing threat was real enough (already in inventory)

| 2012 ^329,330^ | Afghanistan | School water tank | ? no chemical found, but tests reliable? | ?Taliban | School kids; hysteria alleged | ? | 140 to hospital, most mild symptoms, tests all negative and proclaimed hysteria | ? Alludes to other alleged water poisoning incidents at schools in Afghanistan; real gas attacks & threats |
| --- | --- | --- | --- | --- | --- | --- | --- | --- |

<http://www.nydailynews.com/news/crime/oncologist-accused-poisoning-lover-article-1.1367019>

some other salt poisonings of children…

http://www.nj.com/news/index.ssf/2011/02/doctors_scientists_led_heroic.html

Yvonne Goodwin 2008: <http://www.dailymail.co.uk/news/article-1022479/Youre-criminal-Judge-frees-jealous-wife-laced-cheating-husbands-cake-rat-poison.html>

Heather Mook 2007, serial deceit: http://www.dailymail.co.uk/news/article-499516/Embezzler-rat-poison-husbands-spaghetti-cover-43-000-theft.html

…. http://www.dailymail.co.uk/news/article-500134/The-shocking-secrets-conwoman-tried-kill-husband-rat-poison.html

Aletta Elliott 2006, South Africa: http://www.iol.co.za/news/south-africa/rattex-woman-convicted-in-murder-case-1.290788#.VTpXY8t0zcs

Turgut Ozal 1993: http://www.ibtimes.co.uk/turkey-president-poisoned-ozal-murder-400897

Velma 1960s-70s: http://www.nydailynews.com/news/crime/death-row-granny-article-1.378057

Thembisa 2010: http://www.sowetanlive.co.za/news/2010/10/28/boy-in-court-for-poisoning-mom

Cashel Sophia Phinn 2012: http://www.sowetanlive.co.za/news/2010/10/28/boy-in-court-for-poisoning-mom

Leonardo Espinall 2012: http://www.nydailynews.com/news/crime/5-year-old-boy-drowned-father-bronx-police-article-1.1197946

Bavarian man 2012 : http://www.thelocal.de/20121204/46555

German Easter chockie bunny lacer left in his garden 2012: http://www.dailymail.co.uk/news/article-2119222/Pensioner-charged-lacing-chocolate-Easter-bunnies-rat-poison-punish-neighbours-thieving-children.html

Nganjuk East Java Indonesia 2012: http://www.bbc.co.uk/news/world-asia-17054239

Cyanide, Gu Kailai & Neil Heywood 2012: http://www.nytimes.com/2012/08/16/opinion/was-gu-kailai-guilty-of-neil-heywoods-murder.html

Christopher Stevens & Renee Valandingham 2009: http://www.wsaz.com/news/headlines/41745822.html

Ambriorix Burgos 2010: http://bleacherreport.com/articles/445645-breaking-news-former-met-ambiorix-forces-ex-wife-to-eat-rat-poison

Melvin Moore poisoned wife Deborah

Brittany Murphy 2013

James Keown slowly poisoned wife Julie with antifreeze chemical, 2004, for her life insurance (Massachusetts)

William Cain put rat poison in coffee of wife Deborah May 2013

12yo boy something? Liu poisoned himself (tetramine) & 10 classmates in Kunming, Yunnan, 10 Dec 2009 added to water in thermoses, nobody seriously ill

Richard Lyon poisoned Nancy in 1991?

13yo 8^th^ grader puts peanut butter cookies in lunchbox of allergic peer (unharmed, 2008), went to court

Zimbabwe murders: <http://nehandaradio.com/2013/05/08/jealous-husband-kills-6-people-with-poison/>

Xiaoye Wang’s poisoning by thallium, 14 Jan 2011, his wife was prosecuted (daily mail Tianle Li in Feb 2011)

Elisabetta Martini & Marco Coggiola trying to poison relative for money, Pinerolo Italy 2014

Maria Alvarado-Gomez trying to poison self & kids in custody battle, July 2014

Fresno school kids 2012 & Brooklyn school boys 2014 who put rat poison in teacher‘s drink or food

Bury school kids?? 2012 (white board cleaner in teacher drink)

Girl who gave her mother water laced with bleach after an iphone ban (March 2015)

Hal Hill, TN doctor, poisoned wife Liesa with barium in morning coffee for yrs, revealed in 2012

Kisha Jones poisoned pregnant rival (pills) then sent poison milk for the baby, 8 Dec 2009

A few parent poisoning little kid or baby stories I can’t bear to enter

Ana Maria Angulo-gonzalez antifreeze, attempt at ex lover George blumenschein 2014 Houston TX

Robert Ferrante ex-brain surgeon poisoned his wife Autumn Klein with cyanide 2014

Diane and Rachel Staudte antifreeze, 2014 Missouri

Allison Cox used screenwash (in asparagus soup) to poison Jacqueline Gold in 2010: AC claimed it was meant to taste funny not poison: got 12 yr sentence

Florida Pedro Bravo poisoned & suffocated Christian Aquilar, 2012, Florida

Linda Lees poisoned (not killed) Paul Lees 2008: http://www.dailymail.co.uk/news/article-1039610/Wife-poisoned-husband-affair.html

Oscar Pistorius living on tinned food after poisoning threats while in prison, March 2015

Gary Stewart 2010 slug pellets in curry fed to neighbours (feud): <http://www.dailymail.co.uk/news/article-1242637/Gay-man-tried-poison-lesbian-neighbours-slug-pellets-legged-cat-feud-walks-free.html>: acquitted? Presumed he didn’t think it would be harmful??

Sydney woman, 20 Aug 2013, poison put in her coffee by a partner

New Hampshire woman ill on 24-28 March 1999, ex-boyfriend may have had Brucellosis in their flat, why labelled bioterrorism not carelessness??

CIA plots against Fidel Castro, 1960s, as described by Carus, including poison pills that could be dissolved into water (plots only?)

2004 Roman Tsepov, 2007 Yuri Schekochikhin's 2002 Anatoliy Popov, 1999 Anwar Ibrahim, all alleged attacks…

| 2013 ^331^ | London, UK | Diet coke | Abrin | Kuntal Patel | Her mom | Put it in | Only threat | “sparked a huge police operation in London amid fears of a terrorist plot.” |
| --- | --- | --- | --- | --- | --- | --- | --- | --- |
| 2009-10 ^332^ | Orlando FA USA | Food, + soap in water | Rat and insect poison | Michel Pollard (Daughter) | Mother + siblings: Janet Tinoco | ? not clear there was intent to harm | Decline in mother’s health | Not clear, was investigated, girl removed from family & later jailed |
| Sep 1960 ^144^ | Belgian congo | Any, or water | Un-named biological | CIA | Patrice Lumumba | Any thing that goes into mouth | Plot only | Out because single target |
| *Mar 1997 ^144^* | *Akron OH* | *Coca cola* | *Unspecified* | *Neil F. Roberts* | *Coca-cola* | *?* | *Threat only* | *He was convicted for tampering in a plea bargain to lose extortion charge…no threat to health?, so I think this is out* |
| 2008 | Xuzhou City, Jiangsu China | Drinking cups of water | Thallium nitrate | ?firstname? Chang; unclear motive | 3 classmates | Injected | Gravely ill, recovered | ?. |
| 3 Jan 1993 ^333-335^ | Baton Rouge, USA | 1 injected, & 4 had ‘a drink’ | Xylazine, animal tranquilizer | Father = Stanley H. Zukowski; custody dispute | His 5 children | Laced | 5 ill but seems they fully recovered | Arrested at Baton Rouge airport about to fly; booked on 5 counts of attempted murder; pled guilty in May 1993; 12 yr sentence.: *Not include because only 4 targeted victims via food or water* |

Sizzler blackmailer in Sydney NSW, she pretended her food contaminated, no threat was made (so not like the Gerber threats):

| *Jan 2006 ^336^* | *Australia* | *Soup, at salad bar* | *Rat poison* | *(mentally ill indiv)* | *Anyone* | *?* | *No one ill* | *May have been none; Queensland health & police not notified for > 1 month & expressed concerns at restaurant response; police handled otherwise, appears to be complaintant was blackmailer* |
| --- | --- | --- | --- | --- | --- | --- | --- | --- |

| 2009-12 ^337^ | McConnellsburg PA | Drinking water | Visine eyedrops: tetrahydrozoline | Vickie Jo Mills | Thurman Nesbitt (bf) | ? | nausea, vomiting, blood pressure problems and breathing trouble. | His regular Dr. notified authories, police tests… |
| --- | --- | --- | --- | --- | --- | --- | --- | --- |

? Thomas Leahy ^144^: was obsessed with idea of poisoning, talked about putting it into food or water, talked about how it would be easy, got hold of ricin, but never made a threat or had a clear plot that can be described

1984: Canadians Kevin Birch & James Cahoon ^144^: did get hold of Clostridium sub-species, may have wanted to extort by targeting a racehorse, plot was vague at best.

**POOR QUALITY DATA**

Several events in Monterey Institute WMD Terrorism Database that are barely described (no indication of culprit or other details), and cannot be collaborated elsewhere (no independent reference or citation)

Items In Dalziel or other sources which state ‘Not clear if accidental or intentional’ and no other evidence to suggest that event was definitely intentional

**PLAUSIBLE EVIDENCE THAT NOT DELIBERATE CONTAMINATION**

Death of Linda Lin in 1992, not from the food & plausible that baby food not tampered with after all (Dalziel isn’t accurate on this one):

http://www.upi.com/Archives/1991/10/19/Chemists-test-tampered-baby-food/1079687844800/

http://www.nytimes.com/1991/11/16/nyregion/girl-not-killed-by-baby-food-examiner-says.html

http://www.nytimes.com/1991/10/24/nyregion/baby-fed-tainted-food-dies-but-cause-is-unclear.html

Ana Luyong cassava fitters in Philippines (Dalziel & Motarjemi) have this as deliberate) which killed 27 kids & made 100+ ill; poison was a pesticide (coumaphos) easily found when she said maybe the children had cyanide poisoning, many reports to say that prosecutors intended to file charges (she was under emotional stress impersonal life) but no followup whatsoever to confirm charges filed or trial ongoning or conviction, which makes me think that authorities decided that evidence wasn’t strong enough to show it was malicious poisoining. Original thought was she had mistaken coumaphos as flour which fits facts better.

This says that she was charged with reckless homicide (so not deliberate)

http://www.philstar.com:8080/nation/272095/homicide-raps-filed-bohol-poisoning

This report mentions ‘allegedly’ deliberate:

http://www.boholnewstoday.com/201503/mabinis-fallen-27-kids-in-food-poison-10-years-ago-recalled.html

2010 Alexander Clement, 68, and his wife Christine Clement, put sand and salt in jello. News reports say that the couple had no intent to harm, they simply wanted to return for refund (counts as larceny): http://www.cbsnews.com/news/cops-say-couple-tampered-with-pudding-mix/

2012 Brazil indigenous claim: http://www.foxnews.com/world/2012/11/21/brazil-police-investigate-indigenous-claim-that-creek-in-sacred-land-was/

This event left out, because tied to the women’s toilets (kids at this school didn’t drink water from there): <http://www.theguardian.com/world/2006/mar/01/russia.chechnya>

194? Toshima Japan hospital, lots came down with Typhoid

1947-8: cholera outbreaks in Palestine (some tried to blame on Zionists)

2000 Paxon Middle School, Jacksonville FA 2 x 7^th^ graders put rat poison in cafeteria salsa, 34 ill, motive unknown but small amount suggests prank rather than intended harm.

2000: again reads more like prank than intention to actually harm: http://www.spcnetwork.com/mii/2000/001027.HTM

Muriel Morris who prepared lunches for police (fined for violating food hygiene procedures) in 2010

| March 2010 | Stowe School, UK | Carrot & coriander soup | Sanitising destainer = bleach | Maxwell Cook (porter at school) | School kids | ? | No one ingested; he was acquitted of deliberate poisoning although there was a witness |
| --- | --- | --- | --- | --- | --- | --- | --- |

<http://www.nydailynews.com/new-york/nyc-crime/brooklyn-teacher-poisoned-kids-ill-traumatized-article-1.1802944>

thalium accident to 2 ordinary Russians?? In LA: <http://edition.cnn.com/2007/US/03/09/russia.americans.poison/>

Blumenthal’s fat duck closure due to 40 cases of food poisoning, he alleged deliberate sabotage, March 2009

Dumping into water supply of chemicals used to wash out diesel dye on Eire-N.Ireland border, February 2015

Radosavljevic & Belojevic ^338^ give a decent analysis why the alleged Serbian attack on Kosovo water sources (tularaemia) unlikely to be deliberate; BUT they are Serbian authors.

| 6-19 May 1948 ^144,339^ | (Palestine) | Aqueduct from nearby village | Salmonella typhi (Typhoid) | Israeli Nationalist militias | residents of city of Acre | (alleged) via aqueduct into the city; Shaw & MacKay provide plausible alternative explanation | 55 British soldiers & 70 inhabitants ill (3 deaths) | Switched water supplies, vaccination, Chlorination and restricted movement of population; there are similar alleged plots in 1947-48 in Carus |
| --- | --- | --- | --- | --- | --- | --- | --- | --- |

Chongqing Guji kindergarten lesions, not clear deliberate poisoning

| 2014 ^340-342^ | | Higuito de Desamparados and Copalchí de Cartago, Costa Rica | | Water system | | | toxic pesticides and fungicides (found next to supply tank); | False alarm! | | | ? supply for 8000 ppl | |  | Reports of mild illness & hospital visits | | Water service suspended Red Cross, health officials, and police placed on alert; trucks delivering at 6am etc.; big investigation, tests were negative: rumours not sufficient. | |
| --- | --- | --- | --- | --- | --- | --- | --- | --- | --- | --- | --- | --- | --- | --- | --- | --- | --- |
| 2013 | Chongqing, China | | ? | | Rat poison | ? | | | Small kids @ Guji nursery | ? | | 117 got petechiae = blots on skin | | | “Police and health authorities are probing the source of the poisoning”; blood & urine samples sent away | |  |

**INCORRECT AGENT/DELIVERY**

| 1993 ^343^ | Kansas City MO | Pepsico products | ? | Douglas John Knight | Pepsico | ? | Threat only | He was placed under house arrest…not with poison or microbes |
| --- | --- | --- | --- | --- | --- | --- | --- | --- |

Another delivery method planned

MN Patriot’s Council, 1991: 2000 Tucker book: Toxic Terror: Assessing Terrorist Use of Chemical and Biological Weapons (google books)

http://www.sarigordon.com/portfolio/writing-samples/ricin-beans-minnesotans-charged-in-first-domestic-terrorism-arrest/

Moudjaidou Soumanou, Dr Ibrahim Mama Cisse, and Zouberath Kora-Seke in plot on life of Benin President Thomas Boni Yayi (in medicine not food)

**SINGLE OCCASIONAL COMMUNICATED THREAT, WITH NO EVIDENCE OF INTENTION, PLAN OR ABILITY**:

Auckland United Freedom Fighters said they put cyanide in the water; they didn’t and were never heard of again after that single threat: http://www.radionz.co.nz/news/regional/38714/auckland-cbd-water-supply-disrupted-by-cyanide-scare

**REFERENCES**

1. Liu C. Rat Poison Found in Boxed Drinks in Dongguan, One Dead The Nan Fang, Southern Capital Report Sohu News ZGSC. 2015 29 May.

2. Confectionary (Poisoning). HC Deb. 3:30pm ed: UK Parliament; 1984.

3. Algar J. Tainted Homemade Beer Kills 69 in Mozambique. Crocodile Bile to Blame? TechTimes. 2015 12 January.

4. Associated Press. Mozambican Police Arrest Man Suspected of Poisoning Beer. The New York Times. 2015 18 February.

5. Kroll D. Crocodile Bile Expert Suspects Toxic Pesticide In Mozambique Tragedy. Forbescom. 2015 14 January.

6. Beilharz N, Allan T, McAloon C. Blackmailers threaten to poison New Zealand infant milk formula. Australian Broadcasting Corporation. 2015 Mar 10.

7. Rutherford H, Vance A, Moir J, Gulliver A. 'Criminal blackmail threat' to poison baby formula with 1080. NZFarmerconz. 2015 Mar 10.

8. Manhire T. New Zealand prime minister says poison threat to milk powder ‘ecoterrorism’. The Guardian. 2015 20 March.

9. Ryan S. Threat to contaminate infant formula with 1080: Arrest made. New Zealand Herald. 2015 Oct 13.

10. RENMO. Police said the investigation in the dark about tricycle master criminal. RENMOcc. 2015 23 March.

11. Cockroft S. Care worker, 23, charged with attempted murder after 16 residents of home are taken ill with food poisoning-type symptoms. Mail Online. 2014 5 September.

12. Press Association. Care home worker admits attempted murder of colleagues. The Guardian. 2015 20 February.

13. Clarke JS. Is there hope for the child bride accused of murder in Nigeria? . The Guardian. 2014 22 December.

14. AFP. China woman held for nursery poisoning that killed two. Khaleej Times. 2014 Apr 10.

15. Matsalla B. Japan Arrests Suspect in Food Poisoning Scandal Guardian Liberty Voice. 2014 26 Jan.

16. US Department of State. Country Reports on Terrorism 2013. April 2014 2013 (accessed 18 May 2015).

17. Nelson K. Hunan woman poisons 19 children with tainted yogurt. Shangai IST. 2013 21 Dec.

18. National Consortium for the Study of Terrorism and Responses to Terrorism (START). Global Terrorism Database. [Data file]; 2013.

19. British Standards Institute. PAS 96:2014. Guide to protecting and defending food and drink from deliberate attack. London, UK: BSI Standards Limited; 2014.

20. Taylor R. Two schoolgirls die after eating yoghurt 'poisoned by head of rival kindergarten' which was competing for students. The Daily Mail. 2013 May 2.

21. Agence France-Presse. Two sisters die after drinking poison-laced yoghurt in Hebei. The Straits Times. 2013 May 4.

22. Li Y, Gao Y, Yu X, Peng J, Ma F, Nelson L. Tetramine poisoning in China: changes over a decade viewed through the media's eye. *BMC Public Health* 2014; **14**(1): 842.

23. Nicholson G. Another Sad Sandwich. 10 Sept 2013. http://psycho-gourmet.blogspot.co.uk/2013/09/another-sad-sandwich.html (accessed 26 April 2015).

24. rt.com. Bleach plot: Taliban ‘tried to poison’ NATO troops. rtcom. 2012 28 Feb.

25. O'Toole M. Hate crimes unit called in after purported animal-rights group threatens to poison food in Chinatown. National Post. 2011 2 Dec.

26. Jones R. Palestinian admits to poisoning Jewish family Israel Today. 2012 2 September.

27. Hartman B. Workers in poison hummus attack. The Jewish Chronicle Online. 2012 6 September.

28. Kubovich Y. Arab construction workers charged with poisoning Jewish family Haaretz. 2012 2 September.

29. AP. China says milk was tainted with nitrite intentionally the Guardian. 2011 11 Apr.

30. CRIenglish.com. Man captured poisoning food in supermarket 2011 13 January.

31. Herald T. Stunning revelations of deliberate food poisoning at supermarket. 2010 28 October.

32. Keteyian A. Latest Terror Threat in US Aimed to Poison Food. CBS News. 2010 20 Dec.

33. CBS Interactive. Tainted Coffee Poisons Harvard Lab Workers. CBS News. 2009 26 Oct.

34. Gussow L. The Case of the Contaminated Coffee Pot. *Emergency Medicine News* 2010; **32**(1): 8.

35. The Spokesman-Review. Poison ice tea: one more dang thing to worry about. 2015 29 June.

36. BNO News. China sentences three to death for killing tax official, family. Thaindian Times. 2011 11 Nov.

37. Tan K. Kindergarten poisoner sentenced to death. Shanghai Ist. 2011 30 May.

38. Majumdar D. Spurned lover's poisoned curry revenge BBC News. 2010 Feb 10.

39. Investigations FaDAOoC. February 9, 2011: Kansas Woman Sentenced to Prison for Poisoning Salsa at Lenexa Restaurant. US Dept. of Justice; 2011.

40. Environment KDoHa. Outbreaks of Methomyl Poisoning Caused by the Intentional Contamination of Salsa at the Mi Ranchito Restaurant in Lenexa, KS -- August 2009, 2011.

41. US FDA. Manhattan Man Arrested for Posting Threatening Videos on the Internet Claiming to Have Poisoned Baby Food. 2008.

42. Anonymous. The poisoned pickle plot: Retiree tries to blackmail Lidl supermarket chain. Spiegel Online International. 2008 Nov 21.

43. Express.de. http://www.express.de/koeln/er-wollte-millionen-koelner-wegen-lidl-erpressung-verurteilt,2856,820802.html; . Expressde. 2009 Mar 2.

44. The Sydney Morning Herald. At least 100 Afghan officials poisoned. 2008 21 September.

45. US Department of State. Country Reports on Terrorism 2008. April 2009 2008 (accessed 18 May 2015).

46. The Local.SE. Sabotage suspected in mass food poisoning. 2008 26 September.

47. Anonymous. Lidl-Erpresser aus Hessen festgenommen. T-Online. 2008 Aug 20.

48. People's Daily Online. 12 sickened by rice containing rat poison in south China. PDO. 2008 30 June.

49. Dalziel G. Food Defence Incidents 1950–2008. 2009.

50. News24.com. Woman poisoned churchgoers. 2008 30 April.

51. Julian HL. Israeli Intelligence Foils Terrorist Poison Plot in Ramat Ga. IsraelNNcom. 2008 10 April.

52. Li G, Li B, Lin L, et al. [A nitrite poisoning event associated with intentional chemical releases]. *Zhonghua Liu Xing Bing Xue Za Zhi* 2013; **34**(4): 371-3.

53. Ximin H. Food poisoning kills two. Shenzhen Daily. 2008 Feb 25.

54. Anonymous. Tea Bottles Laced With Weedkiller. Japan Probe. 2008.

55. Kwera F. Poisoning attempts scare Kenya refugees. Reuters. 2008 13 Jan.

56. Al-Mashhadani Z, Al-Fatlawy A, Abu Nawas K, Jordan Field Epidemiology Training Program, Al-Nsour M, others a. Thallium Poisoning from Eating Contaminated Cake --- Iraq, 2008. *MMWR Morbidity and mortality weekly report* 2008; **57**(37): 1015-8.

57. Divjak C. Poisoned dumplings incident reveals fragility of Sino-Japanese relations. 3 March 2008. https://www.wsws.org/en/articles/2008/03/dump-m03.html (accessed 15 June 2015).

58. BBC News. Dumplings poisoned 'on purpose' 2008 5 February.

59. Yoshida R. 10 sick after eating tainted ‘gyoza’ from China. The Japan Times. 2008 31 January.

60. Tokyo Weekender. Poisoned frozen gyoza that reached Japan “act of revenge”. 2013 31 July.

61. Associated Press. Chinese dumpling poisoner jailed for life The Guardian. 2014 20 January.

62. People's Daily Online. Government employee commits suicide after killing colleague with rat poison PDO. 2007 Oct 22.

63. Lynn News. Blackmailer jailed. 2008 Jan 21.

64. Lynn News. Food scare at big Lynn store. 2007 Aug 15.

65. The Daily Mail. Conman tried to blackmail supermarket by putting bleach in baby food. The Mail Online. 2008 Jan 22.

66. Orr J, Agencies. Blackmailer jailed over Tesco bomb threats The Guardian. 2008 28 January.

67. Accrington Observer. My brother the troubled Tesco blackmailer. Accrington Observer. 2008 31 January.

68. Marco M. FBI: Kmart Worker Poisoned Meat. The Consumerist. 2007 16 April.

69. Beckenham T. Rat poison strikes in Harbin, Zhejiang and US pet foods. Shanghai ist. 2007 14 April.

70. Wendle J. Serial Poisoner Dies of Poisoning. The Moscow Times. 2008 24 December.

71. Muramila G. Rwanda: Old Woman Held Over Food Poisoning. The New Times: All Africa. 2006 26 Aug.

72. Mercer C. http://www.voanews.com/content/a-13-2006-07-17-voa4/315515.html. 2006 11 Jul.

73. Just-Drinks. SOUTH KOREA: Woman held in Coke "extortion" plot. Just-Drinks. 2006 11 July.

74. AFSPOT. Stolen cyanide 'poisoning enemies'. AFSPOT. 2006 18 May.

75. Radio Australia. Hospital in PNG's Enga province denies cyanide reports. Radio Australia,. 2006.

76. Von Tanja W. http://www.tagesspiegel.de/weltspiegel/lidl-erpressung-ein-fast-perfektes-verbrechen/819568.html. Der Tagesspiegel. 2007 07 Mar.

77. Anonymous. Candeggina in una bottiglia bevuta da un bimbo di 20 mesi. Il piccolo, che ha sputato subito il liquido, è fuori pericolo (Bleach in a bottle drunk by a child of 20 months. The small, which immediately spat the liquid out of danger). La Sicilia. 2004 June 2.

78. Anonymous. Psicosi acquabomber sui succhi di frutta (Psychosis acquabomber on fruit juices). La Nuova di Venezia e Mestre. 2003 Dec 16.

79. Anonymous. Succo di frutta forato, nuovo allarme acquabomber (Juice drilled, new alarm acquabomber ). La Tribuna di Treviso. 2006 Feb 4.

80. Adnkronos. Acquabomber: Succo frutta avvelenato, a donna Palermo finisce in ospedale (Acquabomber: fruit juice poisoned, woman in Palermo ends in hospital). Libro dei Fatti. 2004 Apr 26.

81. Di Mauro A. Acqua contaminata: caso Padova, niente varechina nel formaggio (Contaminated water: Padua case, no bleach in cheese). Agenzia Giornalistica Italia. 2005 Sep 15.

82. Filippi S. Bimba «avvelenata», Acquabomber è innocente (Baby "poisoned", Acquabomber is innocent). il Giornaleit. 2005 Sep 15.

83. NewsOK. No Jail for Spiking Conn. Church Juice NewsOK. 2012 12 July.

84. Team4 News. Man Arrested For Sprinkling Fecal Matter On Pastries. 2005 26 October.

85. AAP General News (Australia). NSW: Australian extortion threats. 2005 19 December.

86. Zhe Z. Rat poison made students vomit. China Daily. 2005 26 Sep.

87. BBC News. Amnesty: West Bank Farms poisoned. BBC News. 2005 25 April.

88. Kruger P. Snickers, Mars Bar recall effort still underway. In: Kruger P, editor. The World Today; 2005.

89. Anonymous. Contaminant in Snickers bar 'similar to pest poison'. The Sydney Morning Herald. 2005 6 July.

90. Glendinning L. Casino is extortion's real target. Sydney Morning Herald. 2005 20 Jul.

91. Maley J, Needham K. Poison threat forces recall of chocolate bars. The Sydney Morning Herald. 2005 2 July.

92. The China Post. Energy drink poisoner is sentenced to death by court. The China Post. 2005 12 July.

93. Taqi A. Man blames wife for poisoning eight children during questioning Gulf News Kuwait. 2005 27 April.

94. Anonymous. Biscuit worker's poison pen bid BBC News. 2006 1 June.

95. Koukouliou V, Ujevic M, Premstaller O. Threats to food and water chain infrastructure: Springer Science & Business Media; 2009.

96. Shetty R. Poisoned Food Sent To Kan. Mayor. CBS News. 2004 2 July.

97. The Nation. I poisoned them, teacher admits. The Nation. 2004 10 July.

98. Fox B, Associated Press. Ricin Found in Jars of Baby Food in California. The Washington Post. 2004 29 July.

99. Bearup G. Girls 'poisoned by militants for going to school' The Guardian. 2004 3 May.

100. Xinhua.

74 ill after eating poisoned food in NW China. China Daily. 2004 15 April.

101. Guan F-y, Liu Y-t, Luo Y, et al. GC/MS identification of tetramine in samples from human alimentary intoxication and evaluation of artificial carbonic kidneys for the treatment of the victims. *J Anal Toxicol* 1993; **17**(4): 199-201.

102. Whitlow KS, Belson M, Barrueto F, Nelson L, Henderson AK. Tetramethylenedisulfotetramine: old agent and new terror. *Ann Emerg Med* 2005; **45**(6): 609-13.

103. China Daily. Farmer poisons children. China Daily. 2003 14 November.

104. Mohtadi H, Murshid AP. Risk analysis of chemical, biological, or radionuclear threats: implications for food security. *Risk Anal* 2009; **29**(9): 1317-35.

105. Associated Press. Rat poison sauce man gets life. The Sydney Morning Herald. 2004 19 July.

106. Farwell J. ‘This light bulb went on’: Nurse recalls identifying arsenic as poison in New Sweden case, 11 years later. Bangor Daily News. 2014 Apr 25.

107. Zernike K. Arsenic Poisoning at Church Mystifies a Maine Town. The New York Times. 2003 1 May.

108. Gensheimer KF, Rea V, Mills DA, Montagna CP, Simone K. Arsenic poisoning caused by intentional contamination of coffee at a church gathering—an epidemiological approach to a forensic investigation. *J Forensic Sci* 2010; **55**(4): 1116-9.

109. Blazek V. Muž s kilem kyanidu skončil ve vazbě. iDNEScz. 2003 29 May.

110. Xinhua News Agency. Falungong Cult Murderer Sentenced to Death. Xinhua. 2003 31 December.

111. Green PS. Czech Republic: Extortionist Wants A.T.M. Cards. The New York Times. 2003 16 May.

112. Velinger J. Court sends cyanide blackmailers to prison. Radio Praha. 2003 7 Nov.

113. Kedroň R. Odvolání kyanidovým vyděračům nepomohlo. iDNEScz. 2004 29 Jan.

114. iDNES.cz. Cyanide blackmailer is in a holding cell. idNEScz. 2003 May 24.

115. iDNES.cz. Vyděrač bere peníze nemocnicím. 2003 16 May.

116. Bradford S. Suspicious wife dished up rat poison to scare husband. South China Morning Post. 2003 18 December.

117. Chau C, AKH L, Tan I. Tetramine poisoning. *Hong Kong Med J* 2005; **11**(6): 511-4.

118. Risen J, Van Natta Jr D. Plot to Poison Food of British Troops Is Suspected. The New York Times. 2003 24 January.

119. MMWR. Nicotine Poisoning After Ingestion of Contaminated Ground Beef --- Michigan, 2003. Centers for Disease Control and Prevention. 2003 8 May.

120. Control CfD, Prevention. Nicotine poisoning after ingestion of contaminated ground beef--Michigan, 2003. *MMWR Morbidity and mortality weekly report* 2003; **52**(18): 413.

121. Control CfD, Prevention. Recognition of illness associated with exposure to chemical agents--United States, 2003. *MMWR Morbidity and mortality weekly report* 2003; **52**(39): 938.

122. en.people.cn. Kindergarten Poisoning Perpetrator Executed in Guangdong. English People China. 2003 3 Jan.

123. Associated Press. China Arrests 2 in School Rat Poisoning 2002 12 December.

124. Croddy E. Rat poison and food security in the People’s Republic of China: focus on tetramethylene disulfotetramine (tetramine). *Arch Toxicol* 2004; **78**(1): 1-6.

125. Haas D. Israeli employers rethink hiring of Arabs / Chef suspected in poisoning plot rattles nerves. Chronicle Foreign Service. 2002 11 September.

126. Arutz Sheva. Jerusalem Cafe Still Employs Arabs Despite Poison Attempt. 2003 11 November.

127. Eckholm E. Man admits poisoning food in rival's shop, killing 38 in China. The New York Times. 2002 Sep 18.

128. News24.com. Rat poison in fatal breakfast. 2002 Sep 16.

129. Associated Press. SPOTLIGHT: Poisoned woman fights to recover. 2012 7 August.

130. Kyodo News International. Man held for allegedly lacing sugar with poison. 2002 28 August.

131. Associated Press. Dutchman convicted for poisoning yoghurt. The Age. 2004 24 March.

132. Chow M. 'Hong Kong bin Laden' admits to poisonings. South China Morning Post. 2002 29 November.

133. BBC News. Vietnamese serial killer on trial. 2004 25 August.

134. AAP General News (Australia). NSW: Man faces court over Sanitarium contamination threats. 2001 11 April.

135. Anonymous. Sins of the son. Sydney Morning Herald. 2004 21 May.

136. Anonymous. Chinese man executed for poisoning classmates 23 Sep 2002 2002. http://deadmaneating.blogspot.co.uk/2002/09/death-from-around-globe-dateline-china.html (accessed 23 September 2015).

137. The Japan Times. Glico weathers extortion demand. 2000 12 August.

138. Howitt AM, Pangi RL. Countering Terrorism: Dimensions of Preparedness: MIT Press; 2003.

139. CBC News. Poison coffee in university machine. 2000 1 June.

140. Thanh Ha T. Coffee arsenic poisoning at university investigated. The Globe and Mail. 2000 2 June.

141. Keremidis H, Appel B, Menrath A, et al. Historical Perspective on Agroterrorism: Lessons Learned from 1945 to 2012. *Biosecurity and bioterrorism: biodefense strategy, practice, and science* 2013; **11**(S1): S17-S24.

142. Edelstein S. Food and nutrition at risk in America: Food insecurity, biotechnology, food safety, and bioterrorism: Jones & Bartlett Learning; 2009.

143. Gowen A. Woman Guilty of Poisoning Will Remain at Crownsville. The Washington Post. 1999 25 November.

144. Carus WS. Bioterrorism and biocrimes: the illicit use of biological agents since 1900: Center for Counterproliferation Research, National Defense University; 2002.

145. Baca K. Outbreak traced to poison. CJOnline. 2002 7 August.

146. Buchholz U, Mermin J, Rios R, et al. AN outbreak of food-borne illness associated with methomyl-contaminated salt. *JAMA* 2002; **288**(5): 604-10.

147. University Wire. Brown Student Poisons Ex-Girlfriend with Iodine-125. The Tech. 1998 17 November.

148. BBC News. Priest killed by 'poisoned chalice'. 1998 12 April.

149. The New Straits Times. Jealous Farmer Poisoned Neighbours. The New Straits Times. 1998 19 November.

150. BBC News. Curry murders suspect swallows nails. BBC News. 2000 30 May.

151. Uede K, Furukawa F. Skin manifestations in acute arsenic poisoning from the Wakayama curry‐poisoning incident. *Br J Dermatol* 2003; **149**(4): 757-62.

152. Asai Y, L Arnold J. Terrorism in Japan. *Prehosp Disaster Med* 2003; **18**(02): 106-14.

153. Tsuchihashi H. Current forensic poison analysis and its value as evidence *Japanese Journal of Forensic Toxicology* 1999; **17**(2): 100-3.

154. Strom S. Possible Cyanide Food Poisoning at Festival Startles Japan. The New York Times. 1998 29 July.

155. Bertrand D. Admits Laced Cookie Plot. New York Daily News. 1997 16 May.

156. AAP. Biscuit extortion case dropped over DNA hurdle. Sydney Morning Herald. 2002 27 Apr.

157. Holloway P, Betts j. A forethought for malice Lawyers Weekly. 2006 Jan 27.

158. Kolavic SA, Kimura A, Simons SL, Slutsker L, Barth S, Haley CE. An outbreak of Shigella dysenteriae type 2 among laboratory workers due to intentional food contamination. *JAMA* 1997; **278**(5): 396-8.

159. Jenkins L. Blackmailer threatened to poison dairy foods. The Times. 1996 28 August.

160. UPI. China executes mother-son poison team. 1996 10 January.

161. UPI. Mother who poisons 18 sentenced to death. 1995.

162. Herald Scotland. Food blackmailer gets four years. Herald Scotland. 2015 Dec 18.

163. Herald Scotland. Aids blackmail man is jailed. Herald Scotland. 1995 Oct 14.

164. Scottish Daily Record & Sunday. Poisoner says God told him to do it. 1996.

165. Associated Press. Vancouver supermarkets pull turkeys after poisoning threat. AP News Archive. 1994 Dec 24.

166. Gadsden Times. Poisoning leaves 15 dead, 12 ill. 1993 4 May.

167. AP. Belgian Student Arrested for Sending Poison Candy. 1992 22 December.

168. Newsday. Belgian tied to poisoning of four in U.S. Reading Eagle / Reading Times. 1992 23 December.

169. Associated Press. Palestinian Poisoned Food, Police Say. The Los Angeles Times. 1992 16 September.

170. UPI Archives. Nearly 800 Chinese poisoned in woman's revenge. 1992 3 July.

171. Associated Press. More Than 500 Poisoned at Chinese School; No Deaths Reported. 1992 21 June.

172. AP. Girl Poisoned to Death by Jealous Stepmother. 1992 18 January.

173. Associated Press. Officials Say Woman's Poisoned By Tainted Soda (sic). 1991 24 August.

174. Associated Press. N.Y. Teacher Jailed in Poisoning Case. The Los Angeles Times. 1992 5 March.

175. Mahoney J. State Police find body in Norwich. TheDailyStarcom. 2013 3 July.

176. Orlando Sentinel. Teacher Gave Colleagues Poison Candy, Police Say. 1991 21 December.

177. UPI Archives. Poisoned snack food sent to lab employees. The Daily Record. 1990 11 April.

178. Associated Press. Extortioners Tampering With British Baby Food. The Los Angeles Times. 1989 27 April.

179. Longworth R. Copycats complicate British baby food case. The Chicago Tribune. 1989 Apr 30.

180. Associated Press. Baby food blackmail hurts three in Britain. The Ocala Star-Banner. 1989 27 April.

181. Lederer EM, Associated Press. Police Hunt Extortionists; Baby Food Tampering Cases Top 200. AP News Archive. 1989 27 April.

182. Motarjemi Y. Encyclopedia of Food Safety: Academic Press Inc 2014.

183. ITN Source. RODNEY WHITCHELO - CONTAMINATING PET FOOD/PLOTTING TO BLACKMAIL HEINZ: [Script], 1990.

184. Lohr S. Baby-Food Makers Confront British Contamination Scare. The New York Times. 1989 2 May.

185. Midgley D. Death row women: The women who are spending their last days on earth on death row. The Daily Express. 2015 28 February.

186. Lethal Injection. Blanche Moore can sometimes look through a thin strip of unpainted window and see birds flying past 31 March 2007 (accessed 12 June 2015).

187. Associated Press. Mother Kills Seven Children and Commits Suicide. 1988 16 December.

188. Desenclos J, Wilder M, Coppenger G, Sherin K, Tiller R, Vanhook R. Thallium poisoning: an outbreak in Florida, 1988. *South Med J* 1992; **85**(12): 1203-6.

189. Wheeler S. After 20 Years in Jail, Alturas Poisoning Killer Still Seeking Appeal. The Ledger. 2011 4 March.

190. Associated Press. Police find third orange juice carton tampered with in Los Angeles area. 1988 July 7.

191. Associated Press. Poisoned Food Adds Twist to Rampage Case. Los Angeles Times. 1988 22 May.

192. AP. Police Still Unraveling Trail Left by Woman in Rampage. The New York Times. 1988 22 May.

193. Papajohn G, Kaplan J, Gibson R, et al. The Many Faces Of Laurie Dann. Chicago Tribune News. 1988 5 June.

194. ASsociated Press. More poisoned candy found in Japan. The Spkesman-Review Spokane Chronicle. 1988 May 27.

195. Suro R. Italy Bans Israeli Grapefruit Sales Amid Scare. The New York Times. 1988 28 April.

196. UPI. Italy orders seizure on poisoned grapefruits. Lodi News-Sentinel. 1988 27 April.

197. Bovsun M. Donald Harvey, serial killer who claims to have murdered 87 in 'mercy killings,' served 15 life sentences. New York Daily News. 2015 24 January.

198. Whalen W. Defending Donald Harvey: The Case of America's Most Notorious Angel-of-Death Serial Killer: Clerisy Press, Emmis Books 2005.

199. McConnell T. Police break silence in hunt for Safeway poisoner. The Glasgow Herald. 1987 15 August.

200. Leubasch AH. Professor pleads guilty in poisoned candy case. The New York Times. 1987 10 June.

201. Kobel P. The Strange Case of the Mad Professor: A True Tale of Endangered Species, Illegal Drugs and Attempted Murder: Globe Pequot Press; 2013.

202. Jones J. MGTOW Tamara Ivanyutina YouTube; 2014.

203. Wakin DJ. Jury Gets Cyanide Tampering Case. Associated Press. 1987 24 September.

204. Associated Press. Police Say Extortionist Put Glass And Poison In Food. 1987 15 August.

205. Katsarelas NG. Ex-Student Charged in Cyanide-Laced Tea Bag Case. Associated Press. 1987 24 March.

206. Janson D. Cyanide death in Jersey called isolated incident. The New York Times. 1986 5 September.

207. KNT News Service. Tests Find Only 1 Soup Packet Had Cyanide. Orlando Sentinel. 1986.

208. Spokesman-Review. Probers seek killer who added cyanide to Lipton Cup-A-Soup. 1986 5 September.

209. Orlando Sentinel. Candy Extortionists. Three people were arrested on charges. 1986 30 May.

210. Sydney Morning Herald. Baby food charge. 1986 19 December.

211. The Age. Man jailed. 1987 16 October.

212. UPI Archives. Guard supermarket turkeys in latest animal-rights scare. 1984 Dec 15.

213. Associated Press. Charge Couple With Trying To Extort Money From Brewery. AP News Archive. 1985 16 January.

214. Mirror SD. Poison sweet plan was an order. 1988 2 February.

215. STAFF. Life In Jail For Poison Doctor. CBSNewscom. 2000 12 July.

216. Stewart JB. Blind Eye: The Terrifying Story Of A Doctor Who Got Away With Murder: Simon & Schuster; 2000.

217. Haberman C. Poisoned Candy Found on Japanese Store Shelves. New York Times. 1984 9 Oct.

218. Anonymous. NPA admits defeat in Glico-Morinaga case. The Japan Times. 2000 10 Feb.

219. Mahoney A. Preventing the Next Attack: An Examination of Policy Issues Brought to Light by the Rajneesh Bioterrorist Attack in Oregon in 1984. *Public Policy in Global Health and Medical Practice Paper, http://policy-csimpp gmu edu/academics/studentpapers/fall2005/fall05_04 pdf* 2005.

220. Flemmer M, Oldfield E. SABOTEURS IN OUR SALAD&quest. *The American journal of gastroenterology* 1998; **93**(2): 278-9.

221. Török TJ, Tauxe RV, Wise RP, et al. A large community outbreak of salmonellosis caused by intentional contamination of restaurant salad bars. *JAMA* 1997; **278**(5): 389-95.

222. UPI Archives. Pesticide found in stuffing mix. 1984 8 May.

223. Martinez I. The history of the use of bacteriological and chemical agents during Zimbabwe's liberation war of 1965-80 by Rhodesian forces. *Third World Quarterly* 2002; **23**(6): 1159-79.

224. Martinez I. Rhodesian Anthrax: The Use of Bacteriological & (and) Chemical Agents during the Liberation War of 1965-80. *Ind Int'l & Comp L Rev* 2002; **13**: 447.

225. UPI. Illinois man accused in 3 arsenic deaths. Bend Bulletin. 1981 20 November.

226. People vs. Albanese. Supreme Court of Illinois 1984. p. 464 N.E.2d 206.

227. Albanese vs. McGinnis. United States District Court; 1993.

228. AP. Tests continue on soft drink after three children poisoned. The Gadsden Times. 1980 31 December.

229. Ledgerwire services. Three children hospitalised in apparent Kool-aid poisoning. The Lakeland Ledger. 1980 30 December.

230. AP. Cyanide wasn't in Kool-aid or sugar. The Lakeland Ledger. 1981 2 January.

231. UPI Archives. An Oregon jeweler serving a 20-year term for a... 1981 23 March.

232. The Washington Post. Extortion by Cyanide. 1980 1 April.

233. AP. Suspect in poison-pickle extortion scheme seized. Reading Eagle. 1980 4 April.

234. UPI. Cancer agent suspected in murders. The Daily Reporter. 1979 13 October.

235. Harper vs. Grammer. United States District Court, D. Nebraska; 1987.

236. JTA. Palestinian Terrorists Inject Mercury into Israeli Oranges; 5 Dutch Children Poisoned After Eating 1978 2 February.

237. Purver RG. Chemical and biological terrorism: the threat according to the open literature: Canadian Security Intelligence Service; 1995.

238. Trends in Japan. Watch What You Eat: Series of Random Poisoning Shocks Japan. 1998 2 November.

239. Reiterman T. Raven: The Untold Story of the Rev. Jim Jones and His People: Tarcher; 2008.

240. anonymous. Velma Margie Barfield. Unknown. http://www.clarkprosecutor.org/html/death/US/barfield029.htm (accessed 22 June 2015).

241. Levin E. Cunning Poisoner—or Redeemed Christian—Velma Barfield Draws Nearer to Her Day of Execution. Peoplecom. 1984 29 October.

242. AP. Man indicted for mail threats. The Lakeland Ledger. 1977 16 July.

243. UPI. Killer resorts to Agatha Christie hint; 4 poisoned. Chicago Tribune. 1978 3 January.

244. Snopes.com. Halloween Poisonings. 31 October 2014 (accessed 8 June 2015).

245. Dexheimer E. 35 years later, memories of notorious Halloween 'Candyman' murder remain vivid. Statesman. 2009 3 November.

246. Kaplan MM, Register DC, Bierman AH, Risacher RL. A nonfatal case of intentional scopolamine poisoning. *Clin Toxicol* 1974; **7**(5): 509-12.

247. Cates W, Silsby HD. Diphenylhydantoin intoxication in a group of military aviators: A case report. *Toxicology* 1973; **1**(4): 377-82.

248. Emsley J. The poison prescribed by Agatha Christie: Thanks to the mystery writer, the deadly properties of thallium sulphate have become common knowledge. The Independent. 1992 20 July.

249. Holden A. St. Albans Poisoner: Life and Crimes of Graham Young Black Swan; 1995.

250. Govern KH. Agroterrorism and Ecoterrorism: A Survey of Indo-American Approaches under Law and Policy to Prevent and Defend against These Potential Threats Ahead. *Florida Coastal Law Review* 2009; **10**: 223.

251. Aquino F. Japan’s Supreme Court rejects retrial request from 1961 killer sentenced to death. Japan Daily News. 2013 17 Oct.

252. Figueiredo Correia Jd. Relatório Figueiredo, 1968.

253. Aggrawal A. Textbook of Clinical and Forensic Toxicology India: Avichal Publishing Company; forthcoming.

254. Trestrail III JH. Criminal poisoning: investigational guide for law enforcement, toxicologists, forensic scientists, and attorneys: Springer Science & Business Media; 2007.

255. The Giggling Granny: Nannie Doss--Serial Killer: Goldmineguides.com; 2015.

256. Murderpedia. Caroline Grills. http://murderpedia.org/female.G/g/grills-caroline.htm (accessed 22 June 2015).

257. Mankowitz ZW. Life between memory and hope: the survivors of the Holocaust in occupied Germany: Cambridge University Press; 2002.

258. Associated Press. Poison Bread Fells 1,900 German Captives In U.S. Army Prison Camp Near Nuremberg. The New York Times. 1946 20 April.

259. Sridharan V. Ebola outbreak: Armed Liberians 'poison' wells killing villagers under pretext of Ebola. International Business Times. 2014 Aug 6.

260. Yates DA. Armed Men Allegedly Poison New Georgia Well. Daily Observer. 2015 8 May.

261. Mendes-Franco J. Oil and Water Don't Mix - Except in Trinidad. Global Voices. 2015 27 Feb.

262. Pearson T. Attacks on Utilities and Other Violence Continues in Venezuela. Venezuelanalysiscom. 2014 27 March.

263. Bloom D. Kindergarten wars: School boss 'put rat poison into water supply of a neighbouring nursery in a twisted bid for business'. The Daily Mail. 2014 23 March.

264. CBC News. U.S. terror suspect studied chemical engineering at Quebec university. Canadian Broadcasting Corporation. 2013 13 May.

265. Gillis W. Was Ahmed Abassi a Via Rail terrorist-in-waiting, or just a talker? The Toronto Star. 2015 12 Mar.

266. Eretz Zen. Rabbit-Killing FSA Terrorists Threaten to Poison Lattakia Drinking Water for an Alawi Genocide (18+) youtube: Eretz Zen; 2012.

267. Wabala D. Kenya: KDF Airlifts Water to Troops as Wells Poisoned. The Star. 2012 24 September.

268. Tampubolon HD. Jakarta Police on alert after food poisoning threat. The Jakarta Post. 2011 14 June.

269. Evans M. Libya: Col Gaddafi troops may have poisoned country's water supply. The Daily Telegraph. 2011 24 Aug.

270. CNA. Suspect detained in water poisoning threat. The China Post. 2009 4 May.

271. Pedersen D. Unknown offenders poisoned the water supply of Mae La refugee camp with weed killer on April 11. 2009.

272. Roul A. Taliban and Weapons of Mass Disruption Threat. CBW Magazine. 2010 January.

273. Burton F. Moscow police warns of plot to poison city water supplies. 2009 (accessed 18 May 2015).

274. Kroll DJ. Securing our water supply: protecting a vulnerable resource: PennWell Books; 2006.

275. Ngozo S. Jealous' man tries to poison community. Times of Swaziland. 2008 16 December.

276. WSAZ. Man Accused of Threatening Water Supply. 2008 8 October.

277. Bakier AH. Jihadis Discuss Means of Poisoning the Water Supply of Denmark and Great Britain. The Jamestown Foundation. 2008 10 September.

278. O'Neill S. Revealed: the ‘terror plot’ to poison water supplies. The Times. 2007 1 September.

279. Leppard D. Al-Qaeda chief planned to poison Britain’s water. *The Sunday Times* 2007.

280. Bianchi S, Bolis R, Dell'Orto C, Lanfranchi E, Zaccone A, Lafranconi A. WHY THE WATER DISTRIBUTION NETWORK SHOULD BE CONSIDIERED CRITICAL INFRASTRUCTURE: European Commission; 2011.

281. Huffman T. Letter-bomb suspect linked to tainted water. Toronto Star. 2007 7 Nov.

282. UPI. Strychnine found in Danish reservoir. 2006 6 October.

283. Hemel Today. WATER SPIKED WITH WEED-KILLER. 2006 10 August.

284. Metrowebukmetro. Scottish terrorists in water threat. Metro Scotland. 2006 11 Sep.

285. Macaskill M, Allardyce J. SNLA threat to poison water supply. The Sunday Times. 2006 10 Sep.

286. ANSA. Toddler victim of bleach poisoning for second time. Italy magazine. 2005 Sep 13.

287. Di Marco M. Milano, torna Acquabomber Tre casi in 24 ore (Milan, Acquabomber is back: Three cases in 24 hours). La Republica. 2004 June 13.

288. Bianchin R. Acquabomber, c'è una banda tutta Italia finisce sotto scacco (Acquabomber is an Italy-wide group, attacks end in checkmate). La Repubblica. 2003 Dec 10.

289. Gregory A. Sabotage scare at Kaikoura reservoir. The New Zealand Herald. 2005 5 May.

290. Kalil J, Berns D. Drinking Supply: Terrorists had eyes on water, 2004.

291. Gleick PH. Water and terrorism. *Water Policy* 2006; **8**(6): 481-503.

292. CNN. Italy on alert for water poisoner. CNNcom. 2003 9 Dec.

293. La Repubblica. Acqua minerale contaminata, casi sospetti in tutta Italia. La Repubblica. 2003 9 Dec.

294. Feuer A. A NATION AT WAR: JORDAN; Iraqi Agents Held in Plot To Poison Water Supply. The New York Times. 2002 2 April.

295. BBC News. China salesman 'poisoned water'. 2003 6 October.

296. Von Derschau V. 'Radicals' arrested near Paris over poison gas attack plot The Independent. 2002 18 December.

297. Peta B. Right-wing plot to poison water foiled. Independent Online News. 2002 25 November.

298. Cameron C. Feds Arrest Al Qaeda Suspects With Plans to Poison Water Supplies. Fox News. 2002 30 July.

299. Associated Press. Earth Liberation Front members threaten Colorado town's water. Billings Gazette. 2002 14 October.

300. Croddy E, Osborne M, McCloud K. Chemical Terrorist Plot in Rome? Monterey Institute of International Studies. 2002.

301. Times Wire Reports. Man Held in Plot to Poison Water. Los Angeles Times. 1999 11 July.

302. Amnesty International. Death penalty / Fear of imminent execution 1999.

303. The Japan Times. Poisonings uncover lax sodium azide controls. 1998 30 October.

304. Wadman M. NIH inquiry fails to find culprit of contamination. *Nature* 1997; **389**(428).

305. Sokolove M. The Food Of A National Institutes Of Health Researcher Was Spiked With Radiation. The Search Is On For A Suspect And A Motive. A Medical Mystery Laden With Criminal Implications. *The Philadelpia Enquirer* 1995.

306. Press Association. 'Safeway poisoner' teaching ethics at Manchester The Guardian. 2004 10 March.

307. The Scotsman. Still haunted by poisoner’s plot. 2002 20 September.

308. The Scotsman. From PhD to ABCs: Safeway tonic poisoner begins literacy crusade. 2002 31 March.

309. McKay R. A gin and tonic laced with Deadly Nightshade..who was trying to poison the capital? Daily Record. 2007 19 October.

310. UPI. Poisoned well kills three soldiers, sickens 25 others. 1993 3 December.

311. Chelyshev A. Terrorists poison water in Turkish Army cantonment. Telegraph Agency of the Soviet Union (TASS), 29 March. 1992.

312. Hunter P. Waterborne Disease: Epidemiology and Ecology. Chichester, England: John Wiley & Sons; 1997.

313. Ramsay C, Marsh J. Giardiasis due to deliberate contamination of water supply. *Lancet (British edition)* 1990; **336**(8719): 880-1.

314. Green P. French team confirms poison in water supply. UPI. 1989 29 December.

315. AP. Paper Says Pretoria Put Germs in Namibian Water. The New York Times. 1990 12 May.

316. AP. Man accused of trying to poison boss. The Tuscaloosa News. 1989 2 July.

317. Orlando Sentinel. Sheep Dip Poisoning. 1987 12 September.

318. Reaves JA. 19 Filipino Police Cadets Killed. Chicago Tribune. 1987 19 September.

319. States S. Security and Emergency Planning for Water and Wastewater Utilities. Denver CO: American Water Works Association 2010.

320. Coates J.

Plot To Poison Water Is Charged: Neo-nazis Targeted Chicago`s Supply, Prosecutors Say. Chicago Tribune. 1988 28 February.

321. Bogen DC, Krey PW, Volchok HL, et al. Threat to the New York City water supply—plutonium. *Sci Total Environ* 1988; **70**: 101-18.

322. AwwaRF project #2981. Standard operating procedures for decontamination of water infrastructure : Investigation into the 1980 Pennsylvania American water incident of purposeful chlordane contamination into its Pittsburgh suburban district's distribution system. 2005 AWWA Water Security Congress. Oklahoma City, OK; 2005.

323. Emmeluth D. Typhoid Fever: Infobase Publishing; 2004.

324. BBC News. Radiation found at 12 locations. BBC News. 2006 Nov 30.

325. BBC News. 'No radiation risk' public told. BBC News. 2006 24 Nov.

326. Harding L. Alexander Litvinenko inquiry: six things we've learned so far. The Guardian. 2015 30 Jan.

327. Shaw K, Anders K, Olowokure B, et al. The international follow-up of individuals potentially exposed to polonium-210 in London 2006. *Public Health* 2010; **124**(6): 319-25.

328. Thompson J, Rehn M, Lossius H, Lockey D. Risks to emergency medical responders at terrorist incidents: a narrative review of the medical literature. Critical care: the Official Journal of the Critical Care Forum; 2014: BioMed Central; 2014. p. 521-.

329. Anonymous. Poisoning shows threat to Afghan girls' education. Deutsche Welle. 2012 18 April.

330. Popalzai M. Poisoned water sickens Afghan boys at school. CNN. 2012 15 May.

331. Peachey P. Daughter cleared of trying to poison mother in ‘Breaking Bad’ plot. The Independent. 2014 2 Oct.

332. Anonymous. Florida Girl Accused of Poisoning Adoptive Family. FoxNews. 2010 29 Oct.

333. The New York Times. Police Say Father of 5 Poisoned His Children. 1993 6 January.

334. Gadsden Times. Man gets 12 years in murder attempt. 1993 16 May.

335. Inquirer Wire Services. Man Guilty Of Trying To Kill His 5 Children The N.j. Vet Had Lost Custody Of The Youngsters. He Gave Them A Heavy Dose Of Animal Tranquilizer. 1993 16 May.

336. TheAge. Woman charged over Sizzler rat poison scare. Theagecomau. 2006 2 Mar.

337. Bonura D. Vickie Jo Mills of McConnellsburg is charged for poisoning boyfriend with Visine drops. The Record Herald. 2012 10 August.

338. Radosavljevic V, Belojevic G. Unusual epidemic events: a new method of early orientation and differentiation between natural and deliberate epidemics. *Public Health* 2012; **126**(1): 77-81.

339. Shaw AB, Mackay H. Double enteric infection (‘la fièvre typhoïde intriquée’). An account of an epidemic. *J Hyg (Lond)* 1951; **49**(2-3): 299-314.

340. Inside Costa Rica. Intentional poisoning of water supply serving 8,000 being described as “terrorism”. Inside Costa Rica. 2014 23 Jul.

341. Ugarte J. Más de 7.000 personas sin agua por aparente contaminación en Desamparados. CRHOY. 2014 22 Jul.

342. Porras Diaz K. Salud descarta contaminación de agua en acueducto de Higuito de Desamparados y Cartago Central de Radios. 2014 23 Jul.

343. Kansas City Star. Man charged in Pepsi extortion case faces trial in Kansas City. The Kansas City Star. 1993 26 Jun;Sect. 3.
